# The *DACH1* gene is frequently deleted in prostate cancer, restrains prostatic intraepithelial neoplasia, decreases DNA damage repair, and predicts therapy responses

**DOI:** 10.1038/s41388-023-02668-9

**Published:** 2023-04-24

**Authors:** Zhiping Li, Xuanmao Jiao, A. Gordon Robertson, Gabriele Di Sante, Anthony W. Ashton, Agnese DiRocco, Min Wang, Jun Zhao, Sankar Addya, Chenguang Wang, Peter A. McCue, Andrew P. South, Carlos Cordon-Cardo, Runzhi Liu, Kishan Patel, Rasha Hamid, Jorim Parmar, James B. DuHadaway, Steven J. M. Jones, Mathew C. Casimiro, Nikolaus Schultz, Andrew Kossenkov, Lai Yee Phoon, Hao Chen, Li Lan, Yunguang Sun, Kenneth A. Iczkowski, Hallgeir Rui, Richard G. Pestell

**Affiliations:** 1 Pennsylvania Cancer and Regenerative Medicine Research Center, Baruch S. Blumberg Institute, 3805 Old Easton Road, Doylestown, PA 18902 USA; 2 Canada’s Michael Smith Genome Sciences Centre, BC Cancer Agency, Vancouver, BC VSZ 4S6 Canada; 3 Lankenau Institute for Medical Research, 100 East Lancaster Avenue, Wynnewood, PA 19096 USA; 4 Division of Perinatal Research, Kolling Institute, Northern Sydney Local Health District, St Leonards, NSW 2065 Australia; 5 Sydney Medical School Northern, University of Sydney, Sydney, NSW 2006 Australia; 6 Department of Cancer Biology, Thomas Jefferson University, Bluemle Life Sciences Building, 233 South 10th Street, Philadelphia, PA 19107 USA; 7 Department of Pathology, Anatomy and Cell Biology, Thomas Jefferson University, Bluemle Life Sciences Building, 233 South 10th Street, Philadelphia, PA 19107 USA; 8 Department of Dermatology and Cutaneous Biology, Thomas Jefferson University, Bluemle Life Sciences Building, 233 South 10th Street, Philadelphia, PA 19107 USA; 9 Department of Pathology, Mt. Sinai, Hospital, 1468 Madison Ave., Floor 15, New York, NY 10029 USA; 10 Abraham Baldwin Agricultural College, Department of Science and Mathematics, Box 15, 2802 Moore Highway, Tifton, GA 31794 USA; 11 Human Oncology and Pathogenesis Program, Marie-Josée and Henry R. Kravis Center for Molecular Oncology, Department of Epidemiology and Biostatistics, Memorial Sloan Kettering Cancer Center, New York, NY USA; 12 Center for Systems and Computational Biology, The Wistar Institute, 3601 Spruce St., Philadelphia, PA 19104 USA; 13 Department of Radiation Oncology, Harvard Medical School, Massachusetts General Hospital, Boston, MA USA; 14 Massachusetts General Hospital Cancer Center, Harvard Medical School, Charlestown, MA USA; 15 Department of Pathology, Medical College of Wisconsin, Milwaukee, WI USA; 16 The Wistar Cancer Center, Philadelphia, PA 19104 USA; 17 Present Address: Dxige Research, Courtenay, BC V9N 1C2 Canada

**Keywords:** Prostate cancer, DNA damage and repair

## Abstract

Prostate cancer (PCa), the second leading cause of death in American men, includes distinct genetic subtypes with distinct therapeutic vulnerabilities. The *DACH1* gene encodes a winged helix/Forkhead DNA-binding protein that competes for binding to FOXM1 sites. Herein, *DACH1* gene deletion within the 13q21.31-q21.33 region occurs in up to 18% of human PCa and was associated with increased AR activity and poor prognosis. In prostate OncoMice, prostate-specific deletion of the *Dach1* gene enhanced prostatic intraepithelial neoplasia (PIN), and was associated with increased TGFβ activity and DNA damage. Reduced *Dach1* increased DNA damage in response to genotoxic stresses. *DACH1* was recruited to sites of DNA damage, augmenting recruitment of Ku70/Ku80. Reduced *Dach1* expression was associated with increased homology directed repair and resistance to PARP inhibitors and TGFβ kinase inhibitors. Reduced *Dach1* expression may define a subclass of PCa that warrants specific therapies.

## Introduction

Prostate cancer (PCa), the second leading cause of cancer-related death in American men, is a genetically heterogeneous disease, likely reflecting distinct genetic drivers [1]. While substratification of PCa into genetic subtypes forms the basis of rational therapy for PCa, current diagnostic tools fail to reliably distinguish aggressive tumors from non-aggressive ones in order to predict therapeutic response [2], and the lack of markers to stratify PCa cases into low- and high-risk groups results in overtreatment of 20–42% of patients [3]. New biomarkers are urgently needed for therapeutic stratification. A better molecular understanding of the disease is necessary to develop novel targeted therapies for PCa, particularly for metastatic PCa.

Defects in DNA damage repair (DDR) pathways are a hallmark of human cancer, with somatic events present in up to 20% of primary PCa [1], including in BRCA2 [4], which participates in homology-directed DNA repair (HR). Defective HR due to defects in BRCA1 or BRCA2 has led to the use of poly(adenosine diphosphate(ADP)-ribose) polymerase (PARP) inhibitors in prostate cancer therapy [5]. Target-region sequencing, array-based gene expression, copy number variation (CNV) analysis, and whole-genome sequencing of tumors have reported several PCa-related genomic alterations, including copy number gains of 8q, and copy number losses of 3p, 8p, 10q, 13q, and 17p [6–8]. In PCa, known genetic drivers for tumor initiation include *PTEN* and *NKX3.1* deletions, rearrangements/fusions of multiple genes (including *TMPRSS2* and the oncogenic ETS transcription factor, *ERG)* [8], and predisposing genetic factors (including germline DNA-repair gene mutations) [9], (reviewed in [1]). Loss of heterozygosity or deletion also occurs within the 13q21 region in PCa, may include BRCA2, and is associated with high-grade prostate cancer [10–12].

In addition to genetic drivers of PCa, hyperactivity of the androgen receptor, inflammation [13], TGFβ activity, and DNA damage contribute to tumor progression [14]. Transforming growth factor β (TGFβ) has tumor‐inhibitory activity in the early stages of prostate tumorigenesis but promotes migration, epithelial-mesenchymal transition (EMT), invasion, and metastasis in late-stage disease [15, 16]. DNA-dependent protein kinase (DNA-PK) is a serine/threonine kinase that, with Ku70, Ku80, XRCC4, ligase IV, and Artemis, drives non-homologous end joining (NHEJ) repair [17]. The heterodimer of Ku70 and Ku80 binds to double-strand breaks (DSBs); it recruits and activates the catalytic subunit DNA-PKC, which in turn recruits the XRCC4/ligase IV heterodimer that is responsible for rejoining the break. Inactivation of the *Ku70* or *Ku80* genes in mice leads to hypersensitivity to radiation, malignant transformation [18, 19], and an associated increase in HR [20, 21], as binding to both ends of a two-ended DSB stabilizes contacts between Ku heterodimers, tethering the DNA ends and preventing access by the HDR machinery [21].

The *Drosophila Dac* gene was initially cloned as a dominant inhibitor of the hyperactive EGFR, *Ellipse* [22]. The human *Dachshund1* (*DACH1*) gene encodes a DNA-binding protein similar to the winged helix/Forkhead subgroup of the helix-turn-helix family. Cyclic amplification and selection of target (CAST), together with ChIP, identified DACH1 DNA binding sequences that resemble Forkhead binding sites [23]. Furthermore, expression of the *DACH1* gene is reported to be reduced in PCa, and DACH1 overexpression inhibited PCa cell line growth [24].

Given the importance of identifying molecular genetic events governing PCa onset and progression, in the work reported here we investigated the role of the *Dach1* gene in PCa progression in transgenic mice. We identify a novel role for DACH1 in maintaining genomic stability through the regulation of DNA repair. A reduced abundance of DACH1 in human prostate cancer, associated with either gene deletion or promoter DNA methylation, correlated with poor outcomes. In prostate OncoMice, prostate-specific deletion of the *Dach1* gene enhanced prostatic intraepithelial neoplasia (PIN), and was associated with increased TGFβ activity and DNA damage. Mechanistically, we show that *DACH1* is recruited to sites of DNA damage, augmenting recruitment of Ku70/Ku80, associated with restraint of homologous end joining (HR). Reduced *Dach1* expression was associated with resistance to PARP inhibitors and TGFβ kinase inhibitors. Our results suggest reduced *Dach1* expression may define a subclass of PCa that warrants specific therapies.

## Results

### The *DACH1* gene is frequently deleted in human prostate cancer (PCa)

**Fig. 1:**
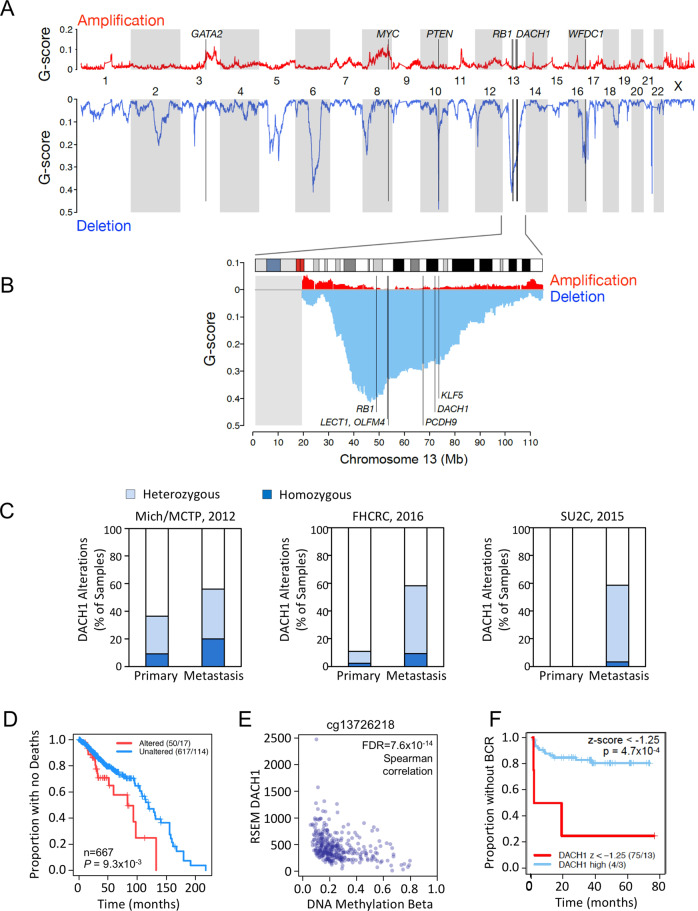
The *DACH1* gene is frequently deleted in human prostate cancer. **A** Landscape of somatic copy number alteration (SCNA) [26] in the TCGA PCa cohort (firebrowse.org) shown as profiles of GISTIC2 G-scores [90] for GRCh37/hg19 SNP6-based data for *n* = 492 primary tumors. (**B**) as from (**A**), for chromosome 13, vertical lines indicate positions of candidate tumor suppressor genes (TSGs) *PCDH9-DACH1-KLF5*. The gray rectangle at the left indicates that TCGA/firebrowse.org reported no GISTIC2 data for the 13p arm. **C** Analysis of *DACH1* gene status in human prostate cancer (PCa) from two cBioPortal cohorts for which copy number data was available for primary and the metastatic sites shows *DACH1* homozygous deletions (dark blue) in 2.3% to 20% of patients and a higher frequency of heterozygous deletions (light blue). **D** DACH1 copy number and overall survival data was determined by combining three cBioPortal cohorts (TCGA PanCancer Atlas 2018, SU2C 2019, and MCTP) (*N* = 667 tumor samples). Kaplan–Meier plot for overall survival is shown using a copy number threshold of −2 to segregate the data into samples with altered vs. unaltered DACH1 [26]. Patients with homozygous *DACH1* deletions (“Altered” in the figure caption) showed reduced overall survival (log-rank *P* = 9.3 × 10^−3^) (“Altered” 50/17 vs. “Unaltered” 617/114). The numbers (50/17, 617/114) indicate the number of samples in the group (e.g., Altered = 50, Unaltered = 617) and the number of events, i.e., for overall survival, deaths (e.g., Altered = 17, Unaltered = 114). **E** The relationship between DNA methylation beta values for probe cg13726218 (reported for *DACH1* at cBioPortal) and RNA-Seq by Expectation Maximization (RSEM) DACH1 normalized expression (*n* = 333 cohort) [26]. The (negative) Spearman correlation between beta and RSEM DACH1 normalized expression was rho = −0.41, FDR = 7.6 × 10^−14^. **F** Kaplan–Meier plot data from Gerhausen et al. [27] showing low DACH1 gene expression (expressed as a z-score with a z-score threshold of −1.25) is significantly correlated with earlier biochemical recurrence (BCR) (log rank *p* value = 4.7 × 10^−4^ (*n* = 79).

In order to determine whether the *DACH1* gene is deleted in PCa we interrogated the genomic sequencing analysis from four cBioPortal cohorts (Mich/MCTP, *N* = 59; Broad/Cornell, *N* = 57; SU2C/PCF (2015), *N* = 150; FHCRC, *N* = 54). Genomic deletion at 13q21 (~18 Mb) has been associated with aggressive prostate cancer [11, 12], although specific genetic drivers were not identified. In recent studies of PCa in a Chinese population, *PCDH9* was noted to be one of the most frequently deleted genes in 13q21.31-q21.33 (~11 Mb) [25]. In TCGA data for 492 primary tumors (firebrowse.org), we identified *DACH1* as a frequently deleted gene within this region (Fig. 1A, B). GISTIC2 analysis identified 51 (10%) ‘deep’ (i.e., homozygous) deletions of *DACH1*. In six distinct cohorts, homozygous deletions of *DACH1* were identified in between 3 and 18% of prostate cancers (Fig. 1C, Supplementary Fig. S1). Median z-scores for DACH1 RNA-seq were progressively larger for DACH1 homozygously deleted, heterozygously deleted, and diploid, indicating that somatic copy number can influence DACH1 expression (Supplementary Fig. S3A). In two cohorts for which CNV data was available for primary and metastatic sites, we identified the relative prevalence of deep and shallow (heterozygous) genomic deletions in both sites (Fig. 1C), finding *DACH1* homozygous deletions more frequent in the metastatic site than in the primary tumors (Mich: 9.1% vs. 20%, *N* = 59; FHCRC: 2.3% vs. 9.3%, *N* = 54). The prevalence of *DACH1* heterozygous deletions was higher in the metastatic lesions than in primary tumors within a given cohort for three of six cohorts (Mich: 27.3% vs. 36%, *N* = 59; FHCRC: 8.5% vs. 48.8%, *N* = 54, respectively) (Fig. 1C).

To assess the relationship between DACH1 homozygous deletions and outcomes, we queried copy number and overall survival data for three cBioPortal cohorts (TCGA PanCancer Atlas 2018, SU2C 2019, and MCTP) (*n* = 667 tumor samples). Using a somatic copy number threshold of −2 to segregate the data into samples with altered *vs*. unaltered DACH1, we generated a Kaplan–Meier plot for overall survival (Fig. 1D) using R’s survival v3.2-7 package, and calculated median times for altered vs. unaltered DACH1 with a custom R script. The patients with homozygous *DACH1* deletions (referred to as “Altered” in the figure caption) had reduced overall survival (medians of 84 vs. 120 months, *N* = 667, log-rank test *P* = 9.3 × 10^−3^) (Altered 50/17, Unaltered 617/114) (Fig. 1D).

*PTEN*-deletion PCa showed infrequent concurrent *DACH1* deletions in 5 of the 6 cohorts (not all datasets are independent), with a strong trend toward mutual exclusivity (Supplementary Fig. S1). In 5 cohorts, the majority of heterozygous *DACH1* deletions were associated with *RB1* heterozygous deletions (Supplementary Fig. S2). However, both *DACH1* homozygous and heterozygous deletions occur in the absence of *RB1* deletions (79/150, 25/444, 13/492), and discordance between *RB1* and *DACH1* genetic deletions was also observed (Supplementary Fig. S2C, D).

In addition to gene deletion, the abundance of DACH1 mRNA may be affected by DNA methylation. We assessed DACH1 DNA methylation and RNAseq expression data from firebrowse.org, for the *n* = 333 primary tumor samples and *n* = 43 adjacent tissue normal samples in the cBioPortal TCGA 2015 cohort [26]. Of 29 Illumina 450 K probes associated with DACH1, 26 had substantially complete data, and 15 of these probes were within ~3 kb of the DACH1 transcriptional start site (TSS) (Supplementary Fig. S3B). Beta values (β) estimate DNA methylation level using the ratio of intensities between methylated and unmethylated alleles, and are continuous variables between 0 and 1, with 0 being unmethylated and one fully methylated. Probe cg13726218, which cBioPortal associates with *DACH1*, had both a wide range of beta values (Supplementary Fig. S3B, probe indicated by a red asterisk) and a strongly-negative Spearman coefficient with DACH1 RNA-Seq expression (rho = −0.41, FDR = 7.6 × 10^−14^) (Fig. 1E). The additional fourteen of the 15 probes in proximity to the TSS had narrow ranges of low beta values in primary tumor samples (Supplementary Fig. S3B). Median beta values were similar in primary tumors and adjacent tissue normals for 25 of the 26 probes (Supplementary Fig. S3C).

The relationship between cg13726218 DNA methylation and gene expression could be expressed as a linear negative correlation (Supplementary Fig. S3; *n* = 308 DACH1-diploid samples, Pearson correlation cor = −0.40, *p* = 2.9 × 10^−13^, alternative hypothesis: true correlation ≠ 0). As a nonlinear alternative, a fit Generalized Additive Model (GAM) had a minimized generalized cross-validation (GCV) score of 0.83 and an adjusted R^2^ of 0.16 (Supplementary Fig. S3E). Collectively, these results indicate that increasing promoter DNA methylation contributed to a reduction in DACH1 mRNA.

As both gene deletion and increasing DNA methylation reduced DACH1 mRNA abundance, we next investigated the potential role of DACH1 gene expression in outcome using SCNA, interrogating data from Gerhausen et al. [27]. Low DACH1 gene expression (expressed as a z-score < −1.25) was significantly correlated with earlier biochemical recurrence (BCR, log-rank *p*-value = 4.7 × 10^−4^, *n* = 79, Fig. 1F). We further assessed RSEM expression for DACH1 (TCGA cohort [26], *n* = 290 of 333 samples). Defining “altered DACH1” samples as those in which DACH1’s RSEM z-score was below −2.0, and using PanCancer outcomes [28], we found a Kaplan–Meier log-rank *P* value of 0.028 for Progression Free Interval (PFI) outcomes (Supplementary Fig. S3F).

Immunostaining for DACH1 protein was conducted in tissue microarrays (TMAs) of 68 cases in triplicate 1 mm biopsy cores of primary prostate cancer with matched normal/benign tissue. DACH1 was positive in all normal/benign prostate epithelia but was negative in 9 of the 68 cases (13.2%). 89% (8/9) of DACH1-negative cases were of high grade (Gleason 4 + 3 = 7 or higher) whereas of DACH1-positive cases only 54% (32/59) were high grade (likelihood-ratio test *p* = 0.034). Representative images of DACH1-positive and -negative cases of prostate cancer are shown in Supplementary Fig. S4.

### *DACH1*-deletion tumors constitute a prostate cancer subtype

**Fig. 2:**
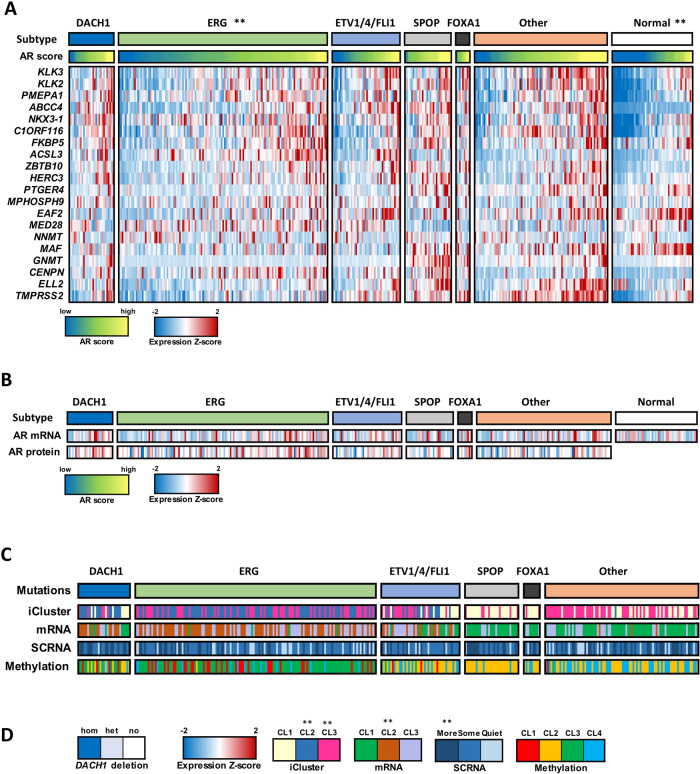
*DACH1* deletion PCa enhances AR signaling. **A** Interrogation of human PCa gene expression data [26], showing candidate genetic drivers *ERG*, *ETV1*/*ETV4*/*FLI1, SPOP, FOXA1*, and unknown. Samples with *DACH1* homozygous (deep) genetic deletions (29/333) are shown as an additional subtype. The AR score (the average of the AR target gene expression) refers to a group of AR-responsive genes [26], and together with the expression Z-score of the AR target genes, are shown as colorimetric scales. The AR score-based gene names are shown. The androgen receptor (AR) activity, inferred by the induction of AR target genes, was increased in *DACH1* homozygous (‘deep’) deletion PCa compared with normal (*P* = 2 × 10^−5^ by *t*-test) and *ERG* mutation groups (*P* = 0.003 by *t*-test). **B** AR mRNA and AR protein levels, shown for each DACH1 deletion sample, were not significantly different. **C** The iCluster [29], mRNA cluster, and SCNA (somatic copy-number alteration), and DNA methylation status are shown for the PCa classified by the corresponding gene deletion subtypes. **D** *DACH1* homozygous deletions were enriched for iCluster 2 and 3 [29], mRNA cluster 2 (*P* = 0.0003 by Fisher exact test, SCNA (“more” somatic copy-number alteration, *P* = 0.0004 by Fisher exact test), but not for DNA methylation.

Using *DACH1* copy number data for samples from The Cancer Genome Atlas (TCGA) [26], we identified a group of 29 of 333 tumors with *DACH1* homozygous deletion (DACH1 subtype, Fig. 2A). In a prior study, androgen receptor (AR) activity varied widely and in a subtype-specific manner, with *SPOP* and *FOXA1* mutant tumors having the highest levels of AR-induced transcripts [26]. Comparison of AR activity levels (using an AR score derived from the expression of AR target genes [26]) showed a significant increase in the *DACH1*-deletion group as compared to normal samples (*P* = 2 × 10^−5^ by *t*-test) and *ERG* mutation groups (*P* = 0.003 by *t*-test) (Fig. 2A). AR activity expressed as a z score for each prostate cancer subtype also showed an increase in the *DACH1* deletion group as compared to normal samples (Supplementary Fig. S5A). However, the levels of AR mRNA and protein were not statistically significantly different (*p* > 0.05) between *DACH1* genotypes (Fig. 2B). Immunohistochemical (IHC) staining of human PCa samples for AR, categorized by DACH1 IHC status, also showed no significant difference based on the DACH1 tumor status (Supplementary Fig. S5B, C, *N* = 64). *DACH1* homozygous deletions were enriched for iCluster 2 and 3 [29], mRNA cluster 2 (*P* = 0.0003 by Fisher exact test), SCNA (“more” somatic copy-number alteration, *P* = 0.0004 by Fisher exact test), but not for DNA methylation (Fig. 2C, D).

The mutation frequencies of SPOP and FOXA1 differed (*P* < 0.05) between DACH1 deleted and diploid before correcting *P* values for multiple testing (Supplementary Fig. S6A). After Bonferroni correcting, only SPOP’s mutation frequency differed (Bonferroni adjusted p-value = 0.012, Supplementary Fig. S6A). In an oncoprint that included mutations and copy number, DACH1 alterations co-occurred with those for SPOP (*q* < 0.001), and ERG’s alterations were mutually exclusive with SPOP’s (*q* = 0.006) (Supplementary Fig. S6B, C). Interrogation of three additional databases revealed no signficant co-occurrence of SPOP abnormalities with DACH1 deletion. (The co-ocurrence of SPOP abnormality with DACH1 deletion was not signficant in the SU2C/PCF Dream Team cohort [30] (log_2_ Odds Ratio 0.510, p value 0.296), FHCRC [31] (Log2 Odds Ratio, 0.234, *p* value 0.55), MCTP [32] (Log2 Odds Ratio 0.65, *p* value 0.56). BRCA2 deletions were identified in 8% of cases, co-occurring significantly with DACH1 deletions (~1/3 of cases, *P* value < 0.001, Supplementary Fig. S6D).

In prior studies, DACH1 competed with FOXM1 binding in ChIP assays conducted in U2OS cells [23], and restrained FOXM1 target gene expression (Cdc25B, CENPA, CENPB, Aurora B kinase, Skp2, and E-cadherin genes). We assessed FOXM1 expression in DACH1 homozygous deletion tumors (Fig. 2A). Using cBioPortal data for the TCGA Firehose Legacy cohort in PRAD primary tumor samples (*n* = 491) in which DACH1 RNA-Seq is low (z ≤ −2.0), FOXM1 expression was increased (*q* < 0.052), and the expression of the FOXM1 target genes CENPA (*q* < 0.05) and AURKB (*q* < 0.05) were also induced; however, the increase in CDC25B (*q* = 0.75), and CENPB (*q* = 1.0) were not statistically significant. SKP2 was not significantly changed and CDH1 was low (*q* << 0.05). As CDH1 is downstream of EMT regulation, other factors may govern CDH1 expression.

Given the association of DACH1 promoter methylation and mRNA abundance in human PCa (Fig. 1E), we determined whether DNA methylation restrained DACH1 expression in cultured PCa cells (Supplementary Fig. S7). The DNA methylase inhibitor 5-Aza-dC (10 μM) induced DACH1 abundance in LNCaP and C4-2 cells (Supplementary Fig. S7, lanes 1 *vs*. 4, and 7 *vs*. 10). Although 5-Aza-dC slightly increased p53 abundance, the 26S proteasome inhibitors MG132 (20 μM) or N-acetyl-L-leucyl-L-leucyl-L-nor leucinal (LLNL) induced a robust increase in p53 abundance (Supplementary Fig. S7).

### *Dach1* deletion in the prostate promotes prostatic intraepithelial neoplasia (PIN)

**Fig. 3:**
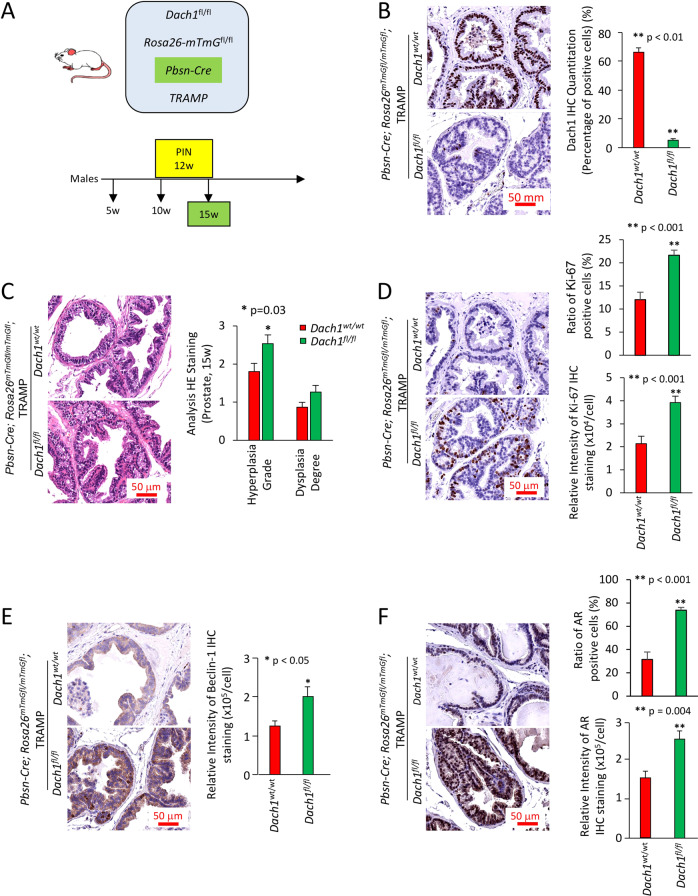
Prostate-specific *Dach1* gene deletion promotes prostate hyperplasia and dysplasia in OncoMice (15 weeks). **A** Schematic representation of transgenes integrated into mice. **B** Representative immunohistochemistry for Dach1, with data quantitated as mean ± standard error of the mean (SEM) for *N* = 20 (4 separate mice, with 5 views per mouse, in each group). **C** Blinded quantitative histology grading of prostate of multigenic mice at 15 weeks. Data are shown as mean ± SEM for *N* = 15 (5 separate mice, with 3 prostate areas [anterior, ventral, lateral] per mouse) in each group). H&E staining demonstrates the presence of a focal atypical intraductal proliferation in *Dach1*^*−/−*^ prostate, compatible with prostatic intraepithelial neoplasia (PIN). Representative immunohistochemistry with results shown as mean ± SEM for Ki-67 (*n* = 20, 4 separate mice for each genotype, 5 views per mouse) (**D**), Beclin 1 (*n* = 9, 3 separate mice for each genotype, 3 views per mouse) (**E**); and AR (*n* = 15 for *Dach1*^*wt/wt*^ mice, 3 separate mice, 5 views per mouse) (*n* = 12 for *Dach1*^*fl/fl*^ mice, 3 separate mice, 2 views for one mouse and 5 views for other two mice) (**F**). Scale bars, 50 μm. A Student’s *t* test was performed for all comparisons.

Given that *DACH1* and *RB1* deletion may co-occur in prostate cancer (Fig. 1B), we sought to determine the functional significance of *DACH1* gene deletion, independently of *RB1*, in the onset and progression of PCa, using a murine model. *Dach1* homozygous null mice die at birth. Given this, in order to determine the role of endogenous *Dach1* in mediating prostate transformation, multigenic mice were generated by crossing conditional *Dach1* gene deletion mice [33] with prostate-specific Cre transgenics, using *Probasin-Cre4* (*Pb-Cre4*) transgenic mice, which express *Cre* in both the basal and luminal prostatic epithelia, then further intercrossing with the TRAMP model of prostate cancer (Fig. 3A). The TRAMP model has been extensively characterized, and TRAMP mice develop PIN after 12 weeks. To follow efficient temporal and spatial regulation of Cre recombination in vivo, we intercrossed these bi-transgenic mice with a double-fluorescent Cre reporter mouse that, prior to Cre-mediated excision, expresses membrane-targeted tandem dimer Tomato (mT) and, after excision, expresses membrane-targeted green fluorescent protein (mG). The mice thus cointegrate four transgenes (Fig. 3A). 3T3 cells derived from *Dach1*^*−/−*^ mouse showed deletion of the *Dach1* gene did not reduce the abundance of pRB (Supplementary Fig. S8). Rather the abundance of pRB^Ser807/811^ was increased by *Dach1* gene deletion when normalized to the protein loading control lamin B1 (Supplementary Fig. S8). Genomic analysis of tail DNA by PCR confirmed the presence of the transgenes in these mice (Supplementary Fig. S9A). Cre-induced GFP was expressed in the prostate epithelium (Supplementary Fig. S9B), whereas no GFP was observed in the absence of Cre recombinase (Supplementary Fig. S9C, D).

Dach1 abundance was identified by immunohistochemistry in the prostate epithelial cells, primarily in the luminal compartment, with additional staining of basal cells in the *Dach1*^wt/wt^ with Probasin-Cre-ROSA26^mTmGfl^-TRAMP mice (shown *Dach1*^*+/+*^) (Fig. 3B). Prostate epithelial cell Dach1 abundance was abrogated in the Probasin-Cre-*Dach1*^*fl/fl*^ ROSA26^mT/mG^-TRAMP line (shown and subsequently referred to as *Dach1*^*−/−*^)(Fig. 3B). In order to determine the role of *Dach1* in the development of murine PIN, *Dach1*^+/+^ and *Dach1*^−/−^ mice were examined at 15 weeks. Based on H&E staining, the prostates of *Dach1*^−/−^ mice showed morphological changes characteristic of PIN, including a larger stromal layer with increased cellularity and nuclear atypia (Fig. 3C). The *Dach1*^−/−^ prostate showed increased prostatic hyperplasia grade (1.8 ± 0.21 vs. 2.5 ± 0.22, *P* = 0.03, *t*-test, *n* = 15, 5 separate mice with 3 areas [ventral, anterior, lateral prostate] per mouse of each genotype) and increased dysplasia degree (0.86 ± 0.13 vs. 1.26 ± 0.17, *P* = 0.08, *t*-test, *n* = 15, 5 separate mice with 3 prostate areas, [vental, anterior, lateral] per mouse in each group) (Fig. 3C). Ki-67 expression is a marker of cellular proliferation and is highly correlated with PIN. The Ki-67 staining intensity was increased in the *Dach1*^−/−^ (Fig. 3D) (2.14 × 10^4^ ± 0.32 × 10^4^ vs. 3.92 × 10^4^ ± 0.27 × 10^4^, *P* = 0.0001, *t*-test) as was the percentage of positive cells (14.10 ± 1.21 vs. 21.60 ± 1.09, *n* = 25, *P* = 0.00026, *t*-test). Beclin 1, an autophagy marker, was increased by >60% (Fig. 3E, *P* < 0.05, *t*-test). AR staining, which was primarily nuclear in the epithelial cells and stroma, was increased in the *Dach1*^*−/−*^ mouse prostate (Fig. 3F, *P* = 0.004, *t*-test). Cleaved caspase 3 was reduced in *Dach1*^*−/−*^ (6.75 ± 1.38 vs. 3.02 ± 0.62, spots per 100 cells, *n* = 5 mice for each genotype, 25 images, *P* = 0.015, *t*-test Supplementary Fig. S10C, D).

### *Dach1* restrains a TGFβ gene expression signaling node in the prostate and in PCa cells

**Fig. 4:**
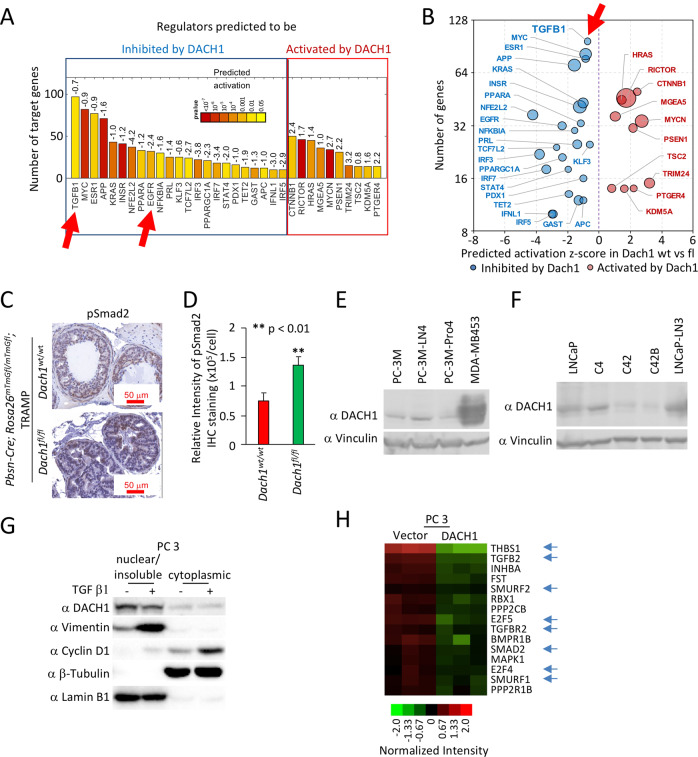
Prostate-specific *Dach1* gene deletion in TRAMP mice induces PIN lesions with increased TGFβ activity. Genome-wide expression analysis of TRAMP *Dach1*^*+/+*^ *vs*. *Dach1*^*−/−*^ PIN lesions was analyzed for enrichment of known targets of upstream regulators using Ingenuity Pathway Analysis (IPA) and represented as (**A**) barplot was calculated by IPA activation Z-score labeled and as (**B**) bubble plot with size of the bubbles proportional to –log_10_ *p* values. **C** IHC was conducted for SMAD activation using SMAD2^P^, quantitated and shown as (**D**) mean ± SEM (*n* = 15 for *Dach1*^*wt/wt*^ mice, 3 separate mice, 5 views per mouse) (*n* = 10 for *Dach1*^*fl/fl*^ mice, 2 separate mice, 5 views per mouse). **E**–**G** Western blot of either PCa cell lines for the presence of DACH1 (**E**, **F**) or (**G**) TGFβ-treated (10 ng/ml for 24 h) PC3 cells illustrating induction of nuclear vimentin and cytoplasmic cyclin D1. Protein loading controls are β-tubulin (a marker of cytoplasmic proteins) and Lamin B1 (a marker for nuclear protein enrichment). **H** Microarray-based gene expression analysis of PC3 cells stably expressing DACH1, showing restraint of genes mediating TGFβ signaling (shown with blue arrows), including reduction of TGFB2 and TGFBR2 [33].

Enrichment analysis of mRNA from ventral prostates from 15-week *Dach1*^+/+^ versus *Dach1*^−/−^ mice identified regulators that were either inhibited or induced by endogenous Dach1 (Fig. 4A, B). TGFβ1 was identified as the gene node with the largest number of target genes repressed by Dach1. Dach1 expression correlated with the inhibition of several additional known pro-proliferative nodes, including c-Myc, ESR1, KRAS, INSR and EGFR (Fig. 4A, B). Consistent with DACH1 impacting TSC2 and Rictor, Ingenuity Pathway Analysis (IPA) showed DACH1 inhibits mTOR signaling (23 genes) *p* = 1.1 × 10^−5^ (Supplementary Fig. S11). In addition, phospho-SMAD2 was increased in the prostate epithelium of *Dach1*^*−/−*^ PIN (Fig. 4C, D; *P* < 0.008), consistent with a role for endogenous Dach1 in reducing TGFβ activity.

Induction of an epithelial-mesenchymal transition (EMT) plays a role in both PCa metastatic progression and resistance to treatment [34]. DACH1 was detectable in human PCa cell lines (Fig. 4E, F), including PC3 cells, in which TGFβ induced EMT, as evidenced by induction of the mesenchymal marker vimentin (Fig. 4G), and an increase in the proportion of cyclin D1 located in the cytoplasm. In addition, PC3 cell lines stably expressing DACH1 showed inhibition of TGFβ target gene expression (Fig. 4H, blue arrows) [33].

### DACH1 governs the DNA damage response to genotoxic stress in PCa cells

**Fig. 5:**
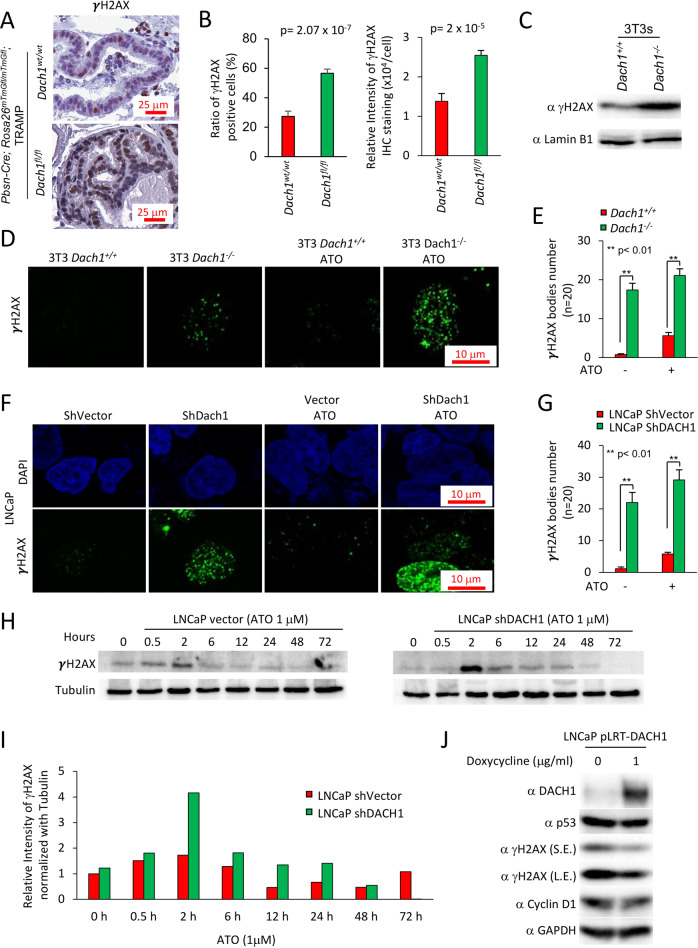
Prostatic *Dach1* governs the DNA damage response in TRAMP mice. **A** Immunohistochemical staining for markers of DNA damage (γH2AX) in TRAMP mice prostates, with (**B**) Quantitation data shown as mean ± SEM for percentage of γH2AX positive cells (*P* = 2.07 × 10^−7^ by Student’s *t* test) (*n* = 19 for *Dach1*^*wt/wt*^ mice, 3 separate mice, 6 views for two mice, 7 views for one mouse) (*n* = 23 for *Dach1*^*fl/fl*^ mice, 4 separate mice, 7 views for two mice, 6 views for one mouse, 3 views for one mouse) (left panel). Data are shown as mean ± SEM for relative intensity of γH2AX (*n* = 16 for *Dach1*^*wt/wt*^ mice, 3 separate mice, 5 views for two mice, 6 views for one mouse) (*n* = 20 for *Dach1*^*fl/fl*^ mice, 4 separate mice, 6 views for two mice, 5 views for one mouse, 3 views for one mouse) (*P* = 2.2 × 10^−5^ by Student’s *t* test) (right panel). **C** Western blot of *Dach1*^*+/+*^ or *Dach1*^*−/−*^ 3T3 cells. **D** γH2AX immunofluorescent staining of *Dach1*^*+/+*^ or *Dach1*^*−/−*^ 3T3 cells, with (**E**) quantitation shown as mean ± SEM, (*n* = 20 separate cells). **F** LNCaP cells transduced with shDACH1, treated with arsenic trioxide (ATO 1 h) (10 μM) and (**G**) quantitation shown as mean ± SEM (*n* = 20 cells). **H** LNCaP cells stably transduced with control vector or shDACH1 were treated with ATO (1 μM) for the time points indicated. Western blotting was conducted for γH2AX, with quantitation of a representative experiment shown in (**I**). **J** LNCaP cell line stably expressing doxycycline-inducible DACH1 were analyzed for the abundance of γH2AX and other proteins as indicated. GAPDH was used as a protein loading control. S.E. short exposure, L.E. long exposure.

γH2AX staining, a marker of the DNA damage response (DDR), was increased by 50% in the *Dach1*^−/−^ mice ventral prostate (Fig. 5A, B) (*P* = 0.00002, Student’s *t* test). The basal level γH2AX abundance was increased 2-fold in *Dach1*^*−/−*^ 3T3 cells (Fig. 5C) with the number of γH2AX bodies increased >15-fold in *Dach1*^*−/−*^ compared with *Dach1*^*+/+*^ 3T3 cells (Fig. 5D, E). Arsenic trioxide (ATO) induces DNA damage through induction of oxidative stress, which mediates cell-cycle arrest and repair of damaged DNA via an ATR/Chk2/Chk1 pathway. ATO increased the number of γH2AX bodies to ~5 in *Dach1*^*+/+*^ and ~20 in *Dach1*^*−/−*^ 3T3 cells (Fig. 5D, E). shDACH1 increased the number of γH2AX bodies in LNCaP cells and enhanced ATO-induced γH2AX bodies from approximately 5 to almost 30 per cell (Fig. 5F, G). The induction of γH2AX in ATO-treated LNCaP cells was further enhanced approximately 2-fold by DACH1 shRNA (Fig. 5H, I). ATO-induced γH2AX abundance remained elevated in DACH1 shRNA transduced cells until 48 h (Fig. 5I). Re-expression of DACH1 with a doxycycline-inducible expression vector in LNCaP cells reduced γH2AX (Fig. 5J).

### DACH1 facilitates the recruitment of, and co-accumulates with, Ku70/Ku80 proteins, at sites of DNA damage

**Fig. 6:**
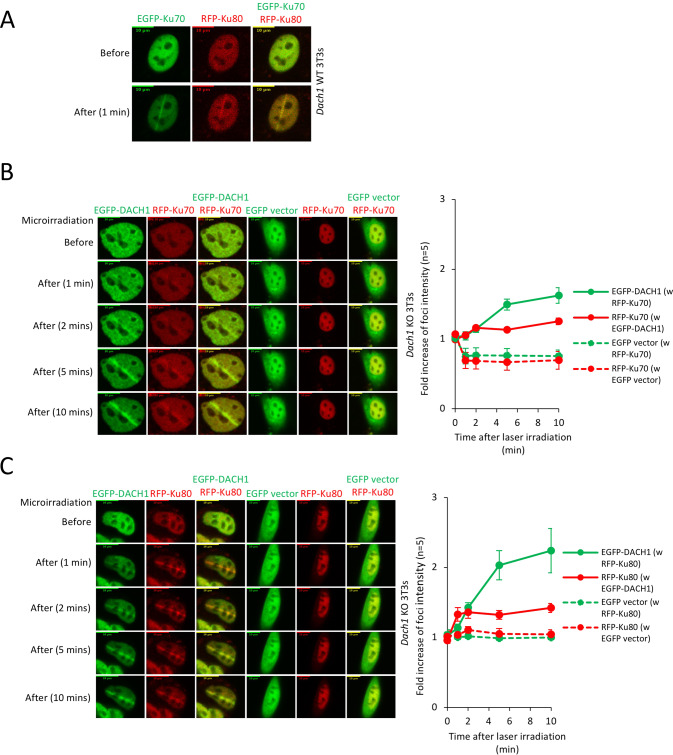
DACH1 facilitates the recruitment of, and co-accumulates with, Ku70/Ku80 proteins at sites of DNA damage. **A** Co-accumulation of Ku-70/Ku-80 at laser micro irradiation-induced DSBs sites in *Dach1*^+/+^ 3T3 cells. **B**, **C** 24 h after transfection, the accumulation of DACH1 and Ku70/Ku80 in *Dach1*^−/−^ 3T3 cells transfected with EGFP or EGFP-tagged DACH1 and red fluorescent protein (RFP)-tagged Ku70 or RFP-tagged Ku80 expression vectors were treated with laser micro-irradiation (403 nm) to induce DSBs. Time is shown after micro-irradiation. Accumulation of the transfected proteins was indicated by EGFP (green) or RFP (red) fluorescence at laser-irradiated sites. Co-accumulation was visualized in yellow merged images. Time is shown in minutes and -fold increase in foci intensity is shown as mean ± SEM for *N* = 5 separate cells.

The Ku70/80 heterodimer is the DNA-binding component of DNA-dependent protein kinase and serves as an early upstream event in NHEJ. In order to determine the functional significance of DACH1 in the recruitment of Ku70/Ku80, we conducted laser micro-irradiation of *Dach1*^*−/−*^ 3T3 cells. In *Dach1*^*+/+*^ 3T3 cells, Ku70 and Ku80 accumulated rapidly at sites of DNA damage after 1 min (Fig. 6A). In *Dach1*^*−/−*^ 3T3 cells, EGFP-DACH1 protein accumulated in ~1 min at sites of DNA damage after laser micro irradiation, and co-localized with RFP-Ku70 or RFP-Ku80 (Fig. 6B, C). Quantitation of protein accumulation at the sites of the laser injury was conducted, and the fold increase in foci intensity is shown as mean ± SEM for *N* = 5 separate cells (Supplementary Fig. S12A–D). *Dach1*^*−/−*^ 3T3 cells transduced with EGFP-DACH1 co-accumulated RFP-Ku70 and RFP-Ku80 at DSB sites (Fig. 6B, C, Supplementary Fig. S12A–D). All three proteins responded to micro irradiation within 1 min, with DACH1 continuing to accumulate at 10 min (Supplementary Fig. S12A–D). In contrast, RFP-Ku70 and RFP-Ku80 failed to localize at the sites of laser irradiation in *Dach1*^*−/−*^ 3T3 cells transduced with the control empty vector construct (Fig. 6B, C, Supplementary Fig. S12A–D).

In order to examine further the interaction between DACH1 and Ku70/Ku80 a mass spectrometry analysis was conducted. DACH1 protein complexes were prepared from HEK 293T cells transfected with a FLAG-DACH1 expression vector (Supplementary Fig. S13A). DACH1-associated proteins were resolved on a 4–10% Tris-HCl gel and silver-stained. The proteins recovered from the gel were subjected to in-gel tryptic digestion and sequential MS/MS. Two excised bands, corresponding to 70 and 80 kDa were identified as ATP-dependent DNA helicase 2 subunit Ku70 and ATP-dependent DNA helicase 2 subunit 2 (Ku80). 293T cells were transfected with expression vectors for FLAG-tagged DACH1 wild type, FLAG-DACH1 DS domain deletion (ΔDS), FLAG-DACH1 C term (Supplementary Fig. S13B). Immune precipitation of FLAG-tagged DACH1 co-precipitated Ku70 and Ku80. Therefore, we considered the possibility that DACH1 may interface with Ku70 via a conserved N-BOX (the *dac* and *ski*/*sno* DS domain) which consists of ∼100 amino acids conserved with various Sno/Ski family members, predicted to form a highly organized structure of α-helices and β-strands referred to as the DS domain. Expression of the DACH mutant deleted of the DS domain (ΔDS) (Supplementary Fig. S13B) abolished binding to Ku70 and Ku80 (Supplementary Fig. S13C). In 293T cells overexpressing FLAG-Ku70 and Myc-DACH1, immunoprecipitation and Western blot analysis with either FLAG-Ku70 or DACH1 (Myc-DACH1) showed binding of DACH1 and Ku70 (Supplementary Fig. S13D).

DACH1 Ser439 is the predominant phosphorylation site and participates in nuclear-cytoplasmic shuttling [35]. Interrogation of PhosphoNET to predict candidate kinases for particular phosphorylation sites [36] identified this motif IKERVPD(ph)SPSPAPSL (pSer439-DACH1) as a potential substrate for several kinases with high proximity scores including JNK1, JNK3, p38 [36] which participate in DNA damage signaling [37, 38], reviewed in [39] (Supplementary Fig. S13E).

### DACH1 decreases homologous DNA repair

**Fig. 7:**
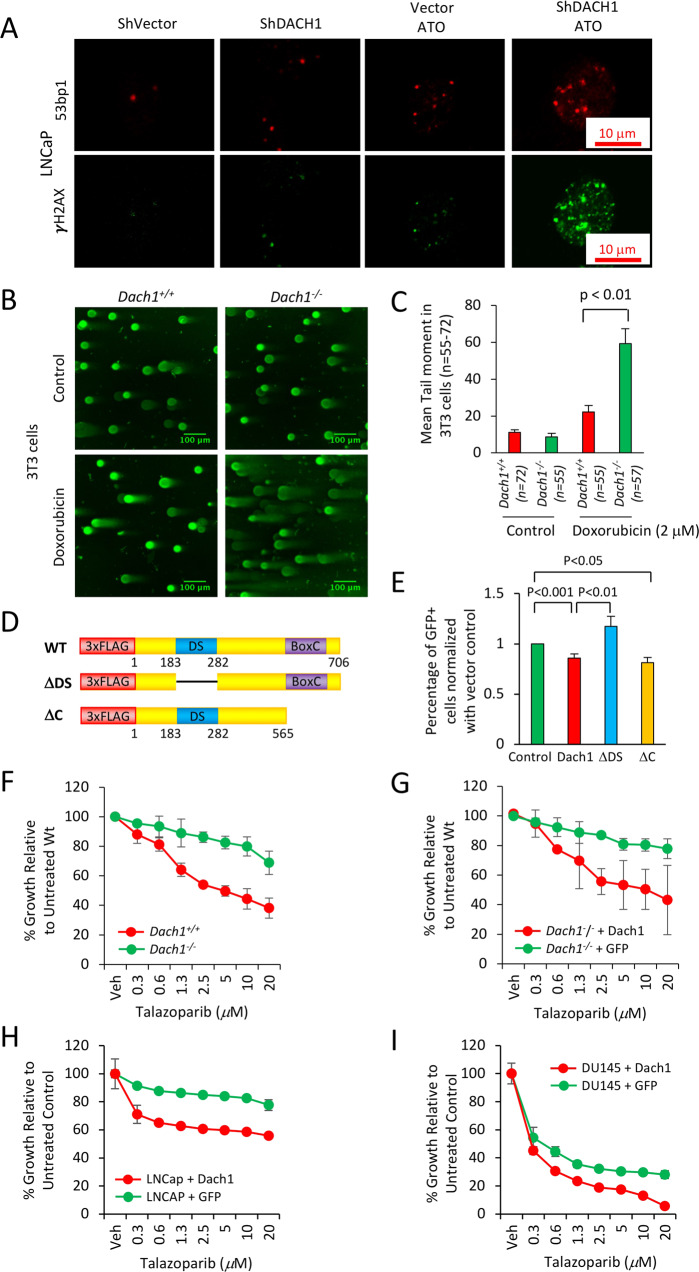
DACH1 enhances DNA repair. **A** LNCaP cells stably transduced with control vector or shDACH1 were treated with ATO (1 μM), and immunofluorescence for 53BP1 or γH2AX was conducted. (**B**) The neutral pH comet assay, which mainly detects DNA double-strand breaks (DSBs), was conducted as a single-cell DNA damage assay. *Dach1*^*+/+*^ and *Dach1*^*−/−*^ 3T3 cells were treated with 2 μM doxorubicin for 18 h. Scale bar, 100 μm with (**C**) data shown as mean ± SEM. **D** Schematic representation of DACH1 expression vectors, which were introduced into (**E**) U2OS cells expressing I-SceI based reporter assays for homologous repair (DR-GFP). Cells were analyzed after 48 h with data shown as mean ± SEM for *N* = 12 (DACH1), *N* = 7 (DACH1 ΔDS), and *N* = 5 (DACH1ΔC). **F** *Dach1*^*+/+*^ and *Dach1*^−/−^ 3T3 cells or (**G**) *Dach1*^−/−^ 3T3 transduced with a MSCV/DACH1-IRES-GFP expression vector or GFP vector control, were treated for 3 days with increasing doses of Talazoparib, a PARP inhibitor. Data are shown as mean ± SEM for triplicate (*N* = 3). **H**, **I** LNCaP (**H**) and DU145 (**I**) cells were transduced with a pLRT-DACH1 expression vector or vector control. After DACH1 induction with doxycycline (1 mg/mL), cells were treated for 3 days with increasing doses of Talazoparib. Cell number was determined by methylene blue assay using a standard curve for each cell line as a reference. Data are shown as mean ± SEM for triplicates (*N* = 3).

Nuclear bodies marked by the DNA damage response protein p53 binding protein 1 (53BP1) [40] participate in the cellular response to DNA damage. 53BP1 relocates to nuclear foci within minutes after exposure of cells to ionizing radiation (IR). Consistent with the finding that endogenous DACH1 governs the DDR, shDACH1 increased basal and ATO-induced 53BP1 nuclear foci formation in LNCaP cells (Fig. 7A).

The comet assay is a surrogate assay for measuring double-stranded DNA breaks in the cell. When cells are electrophoresed in neutral pH, the image looks like a comet with a distinct head composing intact DNA and a tail consisting of damaged DNA, primarily double-strand breaks (DSBs). In doxorubicin-treated 3T3 cells (2 μM, 18 h) the mean comet tail moment was 22 in *Dach1*^*+/+*^ cells, but 59 in *Dach1*^*−/−*^ cells (Fig. 7B, C), indicating that endogenous *Dach1* increases repair of damaged DNA.

To determine the type of DNA repair decreased by Dach1 we deployed I-SceI-based reporter assays for the homology-directed repair (DR-GFP) pathway [41, 42] (Fig. 7D, E). DACH1 expression restrained DR-GFP activity ~15% in U2OS DR-GFP cells (Fig. 7E, *P* < 0.001, *N* = 12). Expression of a DACH1 mutant, deleted of the DS domain, abolished the restraint of DR-GFP activity, whereas expression of a DACH1 mutant deleted of the carboxyl terminus maintained repression of DR-GFP activity ~20% (Fig. 7D, E, *P* < 0.05, *N* = 5). Cell killing with PARP inhibitors (PARPi) was enhanced in cells with reduced homologous repair (HR) [5]. As DACH1 restrained homologous repair based on DR-GFP activity, we investigated the functional significance by assessing the impact of the PARPi Talazoparib. The growth of *Dach1*^*+/+*^ cells was inhibited in a dose-dependent manner (Fig. 7F, T_50_ ~4.5 mM); however, the *Dach1*^−/−^ cells were relatively resistant to PARPi (Fig. 7F, T_50_~1098 mM (projected)). The reintroduction of DACH1 with retroviral-expressing MSCV-DACH1-IRES-GFP into the *Dach1*^−/−^ 3T3 cells restored sensitivity to Talazoparib (Fig. 7G, T_50_ ~10.2 mM) when compared to the *Dach1*^*−/−*^ cells+ GFP vector (T_50_ ~7258 mM (projected)). DACH1 was significantly upregulated in LNCaP and DU145 cells transfected with pLRT-DACH1 in response to doxycycline (1 mg/mL). Overexpression of DACH1 increased PARP inhibitor sensitivity 36 ± 0.13% in DU145 and 46 ± 5.9% in LNCaP at 20 μM Talazoparib (*n* = 3, *p* < 0.005) compared to the vector control cells (Fig. 7H, I).

### DACH1 determines TGFβ response in cell proliferation and DNA damage

Our analysis of the *Dach1*^*−/−*^ TRAMP mice showed Dach1-restrained TGFβ signaling in vivo. In order to determine the role of Dach1 in TGFβ-mediated DNA repair, the TGF-β receptor type I (TGF-βRI) kinase inhibitors (LY2157299 and LY364947) were deployed. *Dach1*^*−/−*^ cells showed enhanced sensitivity to doxorubicin-induced cell-killing that was reversed by the reintroduction of DACH1 (Supplementary Fig. S14A, B). Consistent with hyperactivated TGFβ signaling upon *Dach1* deletion, *Dach1*^*−/−*^ cells were correspondingly resistant to the antiproliferative effect of LY2157299 and LY364947 in the presence of a DNA-damaging agent (Supplementary Fig. S14A, B).

DNA damage repair was assessed by the mean tail movement in comet assays. To avoid an artifact of different cellular proliferation rates we deployed a concentration of LY2157299 and LY364947 in which the antiproliferative effect of the TGFβRki (LY2157299 and LY364947) was similar between *Dach1*^*+/+*^ *vs*. *Dach1*^*−/−*^ 3T3 cells (LY2157299 and LY364947). Pretreatment with LY2157299 and LY364947 for 3 days decreased doxorubicin-induced comet tail formation in *Dach1*^*+/+*^ 3T3 cells (Doxorubicin *vs*. Doxorubicin+LY2157299 or Doxorubicin *vs*. Doxorubicin + LY364947) (Supplementary Fig. S15A–C). Consistent with the resistance of *Dach1*^*−/−*^ to cell killing by TGFBki in the presence of doxorubicin, the TGFβRki did not reduce the DNA damage induced by doxorubicin in *Dach1*^*−/−*^ cells (Supplementary Fig. S15A–C).

In the presence of 4 Gy X-ray irradiation, reduced endogenous DACH1 by shRNA in LNCaP cells, reduced colony formation (survival fraction representing colony number) and colony size (representing cell numbers per colony) in the presence of the DNA-PK inhibitor NU7026 (Supplementary Fig. S16). Endogenous DACH1, therefore, conveyed resistance to NU7026 (Supplementary Fig. S16).

## Discussion

Gene deletion within the 13q21 region has been associated with high-grade prostate cancer and in an independent cohort enriched for early-onset prostate cancer [27] respectively. In the current studies, we identified homozygous deletions of *DACH1* in between 3 and 18% of prostate cancers in six distinct cohorts. *DACH1* is located within the 13q21.31-q21.33 region, which is deleted in poor-prognosis prostate cancer [10–12]. Herein, homozygous *DACH1* deletions correlated with reduced overall survival (medians of 84 vs. 120 months, *N* = 667). *DACH1* deletion co-occurred with deletion of *BRCA2*, consistent with TCGA analysis demonstrating multiple pathways may be disrupted in a given tumor type [43]. The abundance of DACH1 was also reduced by DNA methylation, and low DACH1 gene expression was significantly correlated with earlier biochemical recurrence.

DACH1 is located on chromosome 13q. In a subset of DACH1-deleted prostate cancers, we showed that DACH1 was co-deleted with RB (in 5 databases, co-homozygous-deletion of RB with DACH1 occurred in 15%, 5.3%, 0%, 1.4%, and 9%, Supplementary Fig. S2). The current studies were therefore designed to test the importance of the additional DACH1 deletion in the presence of inactivated RB. Such an approach is necessary to define the additional impact of DACH1 deletion in the onset and progression of PCa. Our studies were therefore designed to distinguish whether DACH1 deletion was an “innocent bystander”, given the genomic proximity of DACH1 to RB. The TRAMP mouse [44–47], one of the most widely accepted in PCa research [48], inactivates pRB via the SV40 Large T antigen, and recapitulates with ~100% penetrance multiple aspects of the human disease, including prostatic intraepithelial neoplasia (PIN) lesions and multifocal invasive carcinoma, and emulates histological and molecular events of human PCa [49]. With time, beyond which our studies were conducted, progression to castration-resistant prostate cancer [50–52], including castrate-resistant PCa (CRPC) [53] and the aggressive therapy-induced neuroendocrine prostate cancer tNEPC [50–52], occurs with metastasis to distant organs [48] including the skeleton [54]. Analysis of the prostates of transgenic mice at 15 weeks demonstrated that genetic deletion of *Dach1* in TRAMP mice correlated with increased Ki-67, histological features of PIN progression, increased TGFβ activity, and increased evidence of DNA damage. An increased rate of PIN in the DACH1 prostate-specific deletion samples (PbCre:Dach1^fl/fl^) was demonstrated by increased cellular proliferation, loss of cellular polarity (black arrow) (Supplementary Fig. S10A, B), nuclear enlargement (white arrow) (Supplementary Fig. S10A, B), and the presence of nucleoli (gray arrow) (Supplementary Fig. S10A, B). Dach1 deletion in the prostate resulted in PIN but not tumorigenesis in the time frame assessed. Thus, deregulation of Dach1 alone, like deregulation of CHD [55], ERG1 [56], or ETV1 [57], is insufficient to drive prostate cancer in the mouse prostate. The genetic disruption of *Dach1* did not disrupt *RB1* gene expression and was associated with enhanced pRB^Ser807/811P^. p-Rb (Rb pSer^807/811^), which serves as a substrate for kinases including cyclin D1/cdk4/6 [58], was reduced by DACH1. DACH1 restrains cyclin D1 in prostate epithelial cells [33] as prostate specific genetic deletion of DACH1 (Dach1^−/−^ PEC) showed increased abundance of cyclin D1 and DACH1 expression in LNCaP cells repressed cyclin D1 abundance [33]. In PCa cell lines, the reintroduction of DACH1 expression reduced RB abundance (Supplementary Fig. S8B). These findings suggest that the effect of *Dach1* deletion in the prostate is not mediated by coincident loss of *RB1*. These results are the first to show that endogenous *Dach1* restrains features of tumorigenesis in vivo, and are consistent with prior correlative studies showing reduced DACH1 abundance in tumors of the brain, ovary, lung, uterus, non-small cell lung cancer, hepatocellular carcinoma, breast, and prostate cancer [24, 59].

In prior tissue culture-based analysis, DACH1 restrained TGFβ signaling by association with NCoR/SMAD4 [60, 61]. The current studies extend these findings by showing that endogenous DACH1 restrains TGFβ signaling in the murine prostate in vivo. TGFβ signaling was the most upregulated pathway by gene expression analysis in the prostate of prostate-specific Dach1-deletion prostate cancer oncomice. TGFβ activity was induced in the Dach1 knockout prostate tissue in vivo as assessed by the induction of SMAD2^Ser465/467P^. Conversely, increased expression of DACH1 in PC3 cells reduced TGFβ activity and expression of TGFβ target genes. In proliferation assays, DACH1 deletion-mediated TGFβ hyperactivation led to relative resistance to the growth-inhibitory effects of the TGFβKRi in the presence of doxorubicin. In the current studies, the inhibition of TGFβ with the TGFβRki (LY2157299) reduced comet tail formation, consistent with recent studies in which *Tgf*β reduced DNA repair and increased comet tail formation in a lung cancer cell line [62]. The reduction in mean tail movement by the TGFbRki (LY2157299) was defective in the *Dach1*^*−/−*^ cells, consistent with increased TGFβ signaling in the *Dach1*^*−/−*^ cells. Increased TGFβ signaling has been strongly linked to PCa [63–65] promoting therapeutic resistance, cell invasiveness and tumor metastasis [66]. The role of TGFβ hyperactivation in DACH1 deletion prostate cancer warrants further analysis.

In the work reported here, DACH1 was shown to promote the repair of damaged DNA. Prostate-specific deletion of the *Dach1* gene in TRAMP mice correlated with increased γH2AX^Ser139^ and 53BP1 staining in prostate tissues and in cultured cells (LNCaP and 3T3). Comet assays of *Dach1*^*−/−*^ cells showed increased tail formation, consistent with defective DNA repair. Analysis of the mechanisms by which DACH1 participates in DNA damage repair identified a chaperone function in which DACH1 augmented the recruitment of Ku70/Ku80 to sites of laser-induced double-stranded DNA breaks. *Dach1* gene deletion reduced recruitment of Ku70/Ku80 to sites of DSB induced by microirradiation, and reintroduction of DACH1 restored Ku70/Ku80 recruitment to DSB. Proteomic analysis of DACH1-binding proteins identified Ku70 and Ku80 by direct sequence analysis and immune precipitation (IP)-Western blotting showed that DACH1 co-precipitated Ku70 and Ku80. *DACH1* overexpressed cells had decreased HR. Like Ku70 or Ku80 deletion cells, reduced DACH1 had increased HR assessed using DR-GFP reporter assays [67]. Herein the restraint of homologous repair by DACH1 required the DS domain. Consistent with the increased HR, *Dach1*^*−/−*^ cells, showed resistance to PARP inhibition. As with *Dach1*^*−/−*^ cells, defective Ku70/Ku80 function is associated with PARP resistance and restoration of HR [68, 69]. Mechanistically, Ku proteins bind to both ends of a two-ended DSB, stabilizing contacts between Ku heterodimers and tethering the DNA ends, thereby preventing access to the HDR machinery [21]. The mechanism by which DACH1 binding to Ku70/Ku80 induces PARP resistance remains to be further determined; however, *Dach1*^*−/−*^ cells appear to be functionally defective in Ku protein recruitment. HR is increased in Ku70 or Ku80 deletion cells [20, 21]. Ku and PARP-1 have been found to compete for binding to DNA ends [70], and PARP1 binds to Ku70/Ku80 [71], blocking its activity [72].

Inactivation of the *Ku70* or *Ku80* genes in mice leads to hypersensitivity to radiation and malignant transformation [18, 19]. Herein, DACH1 shRNA enhanced the radiation sensitivity to DNA-PK inhibition in colony assays. Collectively, these studies show that a loss of Dach1 abundance is associated with resistance to PARPi and TGFβKi, with enhanced sensitivity to radiation and doxorubicin. In addition, a growing body of evidence has identified somatic and, more recently, germline mutations of DNA repair genes in PCa [73]. The ongoing identification and testing of additional genes governing DNA repair may enhance the precision of guided targeted therapies of PCa.

## Materials and methods

### Genetic and epigenetic analysis

To generate profiles of SNP6-based GISTIC2 G-scores along the GRCh37/hg19 human reference genome, and on chromosome 13, we downloaded a ‘scores.gistic’ file for *n* = 492 TCGA legacy PCa (PRAD) primary tumors from firebrowse.org, and an hg19 chromosome length file from genome.ucsc.edu. We generated graphics with a custom R script, and used the R package karyoploteR v1.14.1 to generate the banded chromosome 13 graphic.

To compare *DACH1* copy number gene status in between primary *vs*. metastatic lesions, homozygous and heterozygous deletions in primary and metastatic tumors in different datasets, we used cBioPortal (http://www.cbioportal.org/) [74].

To assess the relationship between DACH1 homozygous deletions and outcomes, we queried mutation, copy number and overall survival data for three cBioPortal cohorts (TCGA PanCancer Atlas 2018, SU2C 2019, and MCTP), which offered data for a total of *n* = 667 tumor samples. Using a copy number threshold of -2 to segregate the data into samples with altered vs. unaltered DACH1, we generated a Kaplan–Meier plot for overall survival using R’s survival v3.2-7 package, and calculated median times for altered vs. unaltered DACH1 with a custom R script.

Comparative analysis of *DACH1* and *PTEN* gene status in all PCa patients and between primary *vs*. metastatic lesions in different datasets was performed using data from cBioPortal (http://www.cbioportal.org/). Selected studies were identified based on query criteria and analyzed using default parameters [74]. Data for analysis of the association between low DACH1 expression with AR activity was derived from the supplementary data of [26], grouped by mutation status of *ERG, ETV1/4/FLI1, FOXA1*, “other” and adjacent tissue normal samples. Additional *DACH1* deletion data for 333 samples from the study were downloaded from cBioPortal [74] and *DACH1* homozygous deletions were defined as a thresholded GISTIC value of −2. All tumor and normal groups were tested for differences in AR score, AR mRNA, and AR protein levels, using two-sample two-tailed Student’s *t* test; results with *p* < 0.05 were considered significant.

To assess the effect of DNA methylation on DACH1 gene expression, we downloaded from firebrowse.org the Illumina 450 K DNA methylation data and RSEM gene expression data for *n* = 498 TCGA ‘legacy’ PCa (PRAD) primary tumors and *n* = 50 adjacent normal tissues. For the 26 DACH1 DNA methylation probes for which DNA methylation data was largely complete, we calculated Spearman correlations between DNA methylation beta values and gene-level RSEM expression in primary tumors with R’s cor.test, then used R’s p.adjust to correct the p-values for multiple hypothesis testing. We assessed scatterplots of DNA methylation beta *vs*. DACH1 gene expression for all 26 DNA methylation probes (data not shown). For each probe, we calculated median beta values for primary tumors and adjacent normal tissue. We compared locations of DNA methylation probes to the DACH1 gene structure by transforming the probe locations into a ‘bed’ file and the Spearman correlation coefficients into a ‘bedgraph’ file (genome.ucsc.edu/FAQ/FAQformat.html), and then displaying both in the UCSC hg19 genome browser (data not shown). This analysis identified that 15 of the 26 DACH1 DNA methylation probes were near that gene’s TSS, including probe cg13726218 (chr13:72438250). This probe had both the largest negative correlation with DACH1 gene expression, and a wide range of beta values (Supplementary Fig. S3A, B). Together, these three factors suggested that this probe represents how DNA methylation can influence DACH1 gene expression, and this was the probe that cBioPortal reported for DACH1.

To assess the relationship between low expression of DACH1 and outcomes, we downloaded DACH1 RSEM expression Z-scores (considering all samples), for the TCGA cohort [26], i.e., for the *n* = 290 of 333 tumor samples that had RSEM data. We defined samples with “altered” DACH1 expression as those in which the gene’s Z-score was below −2.0, which is the default threshold value at cBioPortal. Using the PanCancer outcomes [28], we segregated the samples by low vs not-low RSEM. The R survival v3.2-10 package returned a Kaplan–Meier log-rank *P* value of 0.028 for Progression Free Interval (PFI) outcome data.

For the Firehose Legacy cohort at cBioPortal (*n* = 492 primary tumors), we downloaded thresholded copy number, mutations, and RSEM normalized gene expression data for *DACH1, ERG, ETV1, FOXA1, GBF1, KMT2C, SPOP, TP53*, and *RPRD2*. For *n* = 360 records with homozygous *DACH1* deletions (i.e., CNA = −2, *n* = 51), and diploid *DACH1* (i.e., CNA = 0, *n* = 309), we calculated the percent of mutated samples for *SPOP, FOXA1, KMT2C*, *TP53* and *GBF1* (excluding ERG because it had no mutations in this cohort, and ETV1 because it had only one mutation). For the five retained genes, we calculated *p* values with a Fisher exact test on count data and applied a Bonferroni (x5) correction for multiple hypothesis testing. For *DACH1, ERG, FOXA1, SPOP*, and *RPRD2*, we generated an oncoprint and reported cBioPortal mutual exclusivity and co-occurrence results for pairs of genes. From the RSEM normalized gene expression data, for the *n* = 491 records for which *DACH1* and *SPOPL* had no mutations, we generated a scatterplot of gene expression for these two genes and calculated Pearson and Spearman correlations between the two expression profiles.

### Transgenics

C57BL/6 Dach1^fl/fl^ mice (which remove the same section of the *Dach1* locus that is deleted in the knockout mouse strain [75]) were a kind gift from Dr. Graeme Mardon (Baylor College of Medicine Houston, Texas). C57BL/6 Pbsn-Cre mice which express Cre recombinase under the control of the rat Pbsn (probasin) gene promoter in the prostate epithelium (#026662), C57BL/6 TRAMP mice (Jackson Lab #003135) harbor the Transgenic Adenocarcinoma of the Mouse Prostate (TRAMP) and PB-Tag Line 8247 transgene (#003135) and C57BL/6 ROSA26-mT/mG-flox reporter mice (#007676) were purchased from the Jackson Laboratory. The TRAMP transgenic construct was designed with simian virus 40 (SV40) early genes (large and small tumor antigens, Tag) under the control of the rat probasin promoter. The TRAMP, *Dach1*^*fl/fl*^, Probasin-Cre [33], and ROSA26^mT/mG^ transgenic mice were used to generate a prostate epithelial cell-specific *Dach1* gene knockout mouse (Probasin-Cre-*Dach1*^*fl/fl*^-ROSA26^mT/mG^-TRAMP) lines. The appropriate institutional committee-approved protocols were followed when working with these mice.

### Gross anatomical analysis and immunohistochemistry

Transgenic mice aged 15 weeks were euthanized by CO_2_ asphyxiation. Animals were dissected, and the following organs were removed: ventral, lateral and anterior prostates (AP) for hematoxylin & eosin (H&E) and ventro-dorsolateral (VDL) prostates for immunohistochemical (IHC) staining. Histopathological grading was undertaken in a blinded manner, comparing a total of 10 mice (5 of each genotype), analyzing sections from the anterior, ventral and lateral prostate in each animal (total *N* = 15 in each group). DACH1 (#10914-1-AP, Proteintech), Ki-67 (#M7240, Dako), Beclin 1 (#11427, Santa Cruz Biotechnology), AR (N20) (#sc-816, Santa Cruz Biotechnology), SMAD2^pSer465/467^ (#44-244G, ThermoFisher Scientific), γH2AX (Ser^139^, 05-636, Millipore), Cleaved Caspase-3 (#9661, Cell Signaling Technology), 53BP1 (NB100-304, Novus Biologicals) antibodies were used as described [76]. ImageJ software was used in IHC quantification.

### Microarray analysis

Total RNA was prepared from ventral prostates of 3 *Dach1* WT (Probasin-Cre-*Dach1*^*wt/wt*^ ROSA26^mT/mG^-TRAMP) and 3 *Dach1* KO (Probasin-Cre-*Dach1*^*fl/fl*^ ROSA26^mT/mG^-TRAMP) (15w) using the RNeasy kit from Qiagen following the manufacturer’s instructions (Qiagen). RNA was labeled for hybridization with mouse Clariom D arrays (Applied Biosystems), and analysis was performed as previously described [33, 77]. Gene set enrichment analysis for upstream regulators responsible for a significant number of changed genes was done using QIAGEN’s Ingenuity® Pathway Analysis software (IPA®, QIAGEN Redwood City, www.qiagen.com/ingenuity) with the “Upstream regulator” option or “Canonical pathway” option; regulators or canonical pathways that passed the *P* < 0.01 threshold, had at least ten significantly affected target genes, and had predicted activation states (|Z|> 0.5) were reported.

### DACH1 and AR Immunohistochemical staining

Immunostaining for DACH1 was performed on an Omnis autostainer (Agilent, Santa Clara, CA) on prostate cancer and matched normal/benign tissue in tissue microarray format (triplicate or quadruplicate 1.0 mm punch cores) from prostatectomies of a human cohort of 71 cases [78]. For detection of DACH1 protein, antigen retrieval was done in Tris/EDTA buffer at pH 9 for 30 min at 97 °C, followed by 30 min incubation with rabbit polyclonal DACH1 antibody (Cat. #10914-1-AP, Proteintech, Rosemont, IL; dilution 1:1,000) [33], HRP-conjugated polymer (Envision FLEX, Cat#GV80011-2, Agilent), and DAB chromogen deposition. After cover-slipping, the stained slides were scanned using a bright-field setting on a Pannoramic Flash 250 (3DHistech, Budapest, Hungary). Evaluable DACH1 staining was achieved for 68 cases after the exclusion of cases with missing prostate cancer cores. DACH1 status was considered positive if ≥2% of cancer cells showed detectable staining. Scoring of DACH1 protein expression in prostate cancer cells was performed visually, based on the presence (positive) or absence (negative) of detectable DAB chromogen. Immunohistochemistry for the AR was performed using a Leica Bond Rx autostainer (Leica Biosystems, Deer Park, IL). Deparaffinized sections of prostate TMAs were performed at HpH for antigen retrieval, then incubated with a monoclonal mouse antibody to the Androgen Receptor (Leica Biosystems, catalog #AR-318-L-CE) at 1:50 dilution for 30 min and Bond Polymer Refine Detection kit (Leica Biosystems) was used with DAB (3,3′-Diaminobenzidine) chromogen for visualization. After coverslipping, the slides were scanned using Pannoramic 250 (3DHISTECH Ltd., Budapest, Hungary), and images were generated using Slideviewer software. Likelihood-ratio tests were used for statistical analysis of DACH1 status and Gleason score.

### Cell culture, reagents, and plasmids

HEK293T, PC3, DU145, LNCaP, C4, C4-2 and MDA-MB453 cell lines were originally from the American Type Culture Collection (Manassas, VA). LNCaP-LN3, C4-2B, PC-3M, PC-3M-LN4, PC-3M-Pro4 cell lines were from the University of Texas M.D. Anderson Cancer Center (Houston, Texas). HEK293T, PC3, and DU145 cells were cultured in DMEM supplemented with 10% fetal calf serum, 1% penicillin, and 1% streptomycin. LNCaP, LNCaP-LN3, C4, C4-2, C4-2B, PC-3M, PC-3M-LN4, PC-3M-Pro4, and MDA-MB453 cells were grown as previously described [33, 77]. Mouse embryonic fibroblasts (MEFs) were originally prepared from *Dach1*^*+/+*^ and *Dach1*^*−/−*^ mice [79] and passaged following the 3T3 protocol [61, 80]. The DNA methylase inhibitor 5-Aza-dC (10 μM) (#A3656, Sigma), the 26S proteasome inhibitors MG132 (20 μM) (#S2619, Selleckchem), or N-acetyl-L-leucyl-L-leucyl-L-nor leucinal (LLNL) (25 μM) (#A6185, Sigma), Doxorubicin (Sigma), Talazoparib (SeleckChem), NU7026 (Sigma) were used as described in the text. TGF-β receptor type I (TGF-βRI) kinase inhibitors LY2157299 and LY363947 were bought from Selleckchem (Houston, TX).

The expression vectors encoding DACH1 in CMV10, MSCV-IRES-GFP, or pLRT [35, 81], Ku70 (RFP-Ku70) and Ku80 (RFP-Ku80) [82], shDACH1 [83] were previously described. The EGFP-DACH1 expression plasmid was made by inserting the human DACH1 cDNA into the HindIII and BamHI sites of the pEGFP-C1 vector.

*Dach1*^*−/−*^ 3T3 cells, stably transduced with MSCV-DACH1 or MSCV-GFP control cells, were prepared by retroviral infection as described previously [84]. LNCaP and DU145 prostate cancer cell lines with Tet-inducible DACH1 overexpression were prepared as previously described [35, 81]. Briefly, LNCaP and DU145 were tranduced with pLRT-DACH1 or pLRT vector control with Lipofectamine 2000 (Thermo Scientific) according to the manufecture’s instructions. Two days later cells were selected with 10 μg/ml blasticidin (InvivoGen) for three weeks in the medium with 10% tetracycline free FBS.

### Western blot analysis

Whole-cell lysates, nuclear lysates, or cytoplasmic lysates were separated by 8–11% SDS-PAGE gel and the proteins were transferred to a nitrocellulose membrane for Western blotting, as previously described [33, 77]. The bands were detected using the enhanced chemiluminescence detection system (Thermo Fisher Scientific #34578). The following antibodies were used: DACH1 (#10914-1-AP, Proteintech), γH2AX (#05-636, Millipore, or #80312, Cell Signaling Technology), Vimentin (#5741, Cell Signaling), cyclin D1 (sc-20044, Santa Cruz Biotechnology), p53 (#sc-6243, Santa Cruz Biotechnology), Vinculin (#V9131, Sigma), β-Actin (#sc-47778, Santa Cruz Biotechnology), β-Tubulin (#sc-9104, Santa Cruz Biotechnology), Lamin B1 (#ab16048, Abcam), and GAPDH (#sc-25778, Santa Cruz Biotechnology).

### Immunoprecipitations and Western blotting

HEK293T cells were transiently transfected with DACH1 and/or Ku70 expression vectors or vector control by calcium phosphate precipitation. Two days later cells were lysed in ice-cold immunoprecipitation (IP) buffer [85] (50 mM Tris-HCL at pH 7.4, 50 mM KCl, 5 mM EDTA, 1% IGEPAL CA630, 10% glycerol, 1 mM sodium orthovanadate and protease inhibitor cocktail (Thermo Scientific). For each IP, 1 ml of lysate (1 mg protein) and 20 µl of anti-FLAG M2-agarose affinity gel (Sigma, St. Louis, MO) were incubated overnight at 4 °C. Immunoprecipitates were washed 5 times in IP buffer, and 20 µl of 2 × sample buffer was added to the bead pellet. The immunoprecipitates, as well as 50 µg of proteins from the corresponding lysates were subjected to Western blotting as previously described [33, 77]. The following antibodies were used: rabbit polyclonal DACH1 antibody (Proteintech 10914-1-AP), rabbit polyclonal Ku80 antibody (Invitrogen PA517454), mouse monoclonal Ku70 antibody (Fisher MS329P), mouse monoclonal FLAG antibody (Sigma F3165), and rabbit polyclonal GAPDH antibody (sc-25778).

### Cell proliferation and comet assays

Cells were seeded into 96-well plates in normal growth medium, and cell growth was measured daily by methylene blue assay [86]. Neutral pH comet assays were conducted as previously described [87] using the CometAssay Kit (Trevigen). After treatment with 2 μM doxorubicin or control for 18 h, cells were harvested and mixed with low-melting temperature agarose. After lysis, electrophoresis was conducted at 1 V/cm for 20 min. Visualization involved SYBR Gold dye for 30 min and a Nikon C2 + Confocal Microscope with a 20× objective. Average tail moments from 55 to 72 cells per sample were obtained using OpenComet software (http://www.cometbio.org/index.html) [88].

### Laser micro-irradiation

Cells were transfected with expression vectors encoding fusion proteins of DACH1 (EGFP-DACH1), Ku70 (EGFP-Ku70 or RFP-Ku70), or Ku80 (RFP-Ku80) [82], and, after 24 h, were treated with 100 μM 8-methoxypsoralen and subjected to 405 nm laser irradiation as previously described [82].

### Identification of DACH1-associated proteins by mass spectrometry

The DACH1 complex was purified from HEK 293T cells transiently transfected with expression vectors encoding FLAG-tagged DACH1, following the protocol described in Technical Bulletin (No. MB-925, Sigma-Aldrich, St. Louis, MO). Proteins were digested by the addition of 25 ng/μl sequence-grade modified trypsin (Promega, Madison, WI) in ammonium bicarbonate buffer for 16 h at 30 °C, with agitation. The digestion products were applied onto a MALDI plate as described [89].

### DNA repair assays

The DNA repair reporter assays for homologous repair (DR-GFP) in U2OS cells were conducted as previously described [41, 42]. pCAGGS-NZEGFP, a plasmid encoding expressed GFP, was a transfection efficiency control. The DNA repair activity was shown as (R_SceI_-R_pCAGGS_)/R_NZEGFP_. R_I-SceI_, R_pCAGGS_, and R_NZEGFP_ represent the ratio of GFP positive cells in I-SceI, pCAGGS-BSKX (vector control for I-SceI expression plasmid), and NZEGFP transfected cells respectively.

### Statistical analysis

The statistical significance of mean differences was determined with two-tailed Student’s *t* tests. The statistical significance of two sample proportions was determined with two-tailed two-sample *z*-tests.

## Supplementary information

Supplemental Figures

Supplemental Figure Legends

## References

[1] Wang G, Zhao D, Spring DJ, DePinho RA (2018). Genetics and biology of prostate cancer. Genes Dev.

[2] Dimakakos A, Armakolas A, Koutsilieris M (2014). Novel tools for prostate cancer prognosis, diagnosis, and follow-up. Biomed Res Int.

[3] Draisma G, Etzioni R, Tsodikov A, Mariotto A, Wever E, Gulati R (2009). Lead time and overdiagnosis in prostate-specific antigen screening: importance of methods and context. J Natl Cancer Inst.

[4] Li S, Silvestri V, Leslie G, Rebbeck TR, Neuhausen SL, Hopper JL (2022). Cancer risks associated with BRCA1 and BRCA2 pathogenic variants. J Clin Oncol.

[5] Teyssonneau D, Margot H, Cabart M, Anonnay M, Sargos P, Vuong NS (2021). Prostate cancer and PARP inhibitors: progress and challenges. J Hematol Oncol.

[6] Baca SC, Prandi D, Lawrence MS, Mosquera JM, Romanel A, Drier Y (2013). Punctuated evolution of prostate cancer genomes. Cell.

[7] Shen MM, Abate-Shen C (2010). Molecular genetics of prostate cancer: new prospects for old challenges. Genes Dev.

[8] Tomlins SA, Rhodes DR, Perner S, Dhanasekaran SM, Mehra R, Sun XW (2005). Recurrent fusion of TMPRSS2 and ETS transcription factor genes in prostate cancer. Science.

[9] Pritchard CC, Mateo J, Walsh MF, De Sarkar N, Abida W, Beltran H (2016). Inherited DNA-repair gene mutations in men with metastatic prostate cancer. N Engl J Med.

[10] Dong JT, Boyd JC, Frierson HF (2001). Loss of heterozygosity at 13q14 and 13q21 in high grade, high stage prostate cancer. Prostate.

[11] Chen Y, Sadasivan SM, She R, Datta I, Taneja K, Chitale D (2020). Breast and prostate cancers harbor common somatic copy number alterations that consistently differ by race and are associated with survival. BMC Med Genomics.

[12] Chen C, Brabham WW, Stultz BG, Frierson HF, Barrett JC, Sawyers CL (2001). Defining a common region of deletion at 13q21 in human cancers. Genes Chromosomes Cancer.

[13] Gandaglia G, Zaffuto E, Fossati N, Cucchiara V, Mirone V, Montorsi F (2017). The role of prostatic inflammation in the development and progression of benign and malignant diseases. Curr Opin Urol.

[14] Nelson WG, De Marzo AM, Isaacs WB (2003). Prostate cancer. N Engl J Med.

[15] Batlle E, Massague J (2019). Transforming growth factor-beta signaling in immunity and cancer. Immunity.

[16] Song B, Park SH, Zhao JC, Fong KW, Li S, Lee Y (2019). Targeting FOXA1-mediated repression of TGF-beta signaling suppresses castration-resistant prostate cancer progression. J Clin Invest.

[17] Davis AJ, Chen BP, Chen DJ (2014). DNA-PK: a dynamic enzyme in a versatile DSB repair pathway. DNA Repair (Amst).

[18] Difilippantonio MJ, Zhu J, Chen HT, Meffre E, Nussenzweig MC, Max EE (2000). DNA repair protein Ku80 suppresses chromosomal aberrations and malignant transformation. Nature.

[19] Smith GC, Jackson SP (1999). The DNA-dependent protein kinase. Genes Dev.

[20] Pierce AJ, Hu P, Han M, Ellis N, Jasin M (2001). Ku DNA end-binding protein modulates homologous repair of double-strand breaks in mammalian cells. Genes Dev.

[21] Stark JM, Pierce AJ, Oh J, Pastink A, Jasin M (2004). Genetic steps of mammalian homologous repair with distinct mutagenic consequences. Mol Cell Biol.

[22] Mardon G, Solomon NM, Rubin GM (1994). dachshund encodes a nuclear protein required for normal eye and leg development in Drosophila. Development.

[23] Zhou J, Wang C, Wang Z, Dampier W, Wu K, Casimiro MC (2010). Attenuation of Forkhead signaling by the retinal determination factor DACH1. Proc Natl Acad Sci USA.

[24] Wu K, Katiyar S, Witkiewicz A, Li A, McCue P, Song LN (2009). The cell fate determination factor dachshund inhibits androgen receptor signaling and prostate cancer cellular growth. Cancer Res.

[25] Ren S, Wei GH, Liu D, Wang L, Hou Y, Zhu S (2018). Whole-genome and transcriptome sequencing of prostate cancer identify new genetic alterations driving disease progression. Eur Urol.

[26] Cancer Genome Atlas Research N. (2015). The molecular taxonomy of primary prostate cancer. Cell.

[27] Gerhauser C, Favero F, Risch T, Simon R, Feuerbach L, Assenov Y (2018). Molecular evolution of early-onset prostate cancer identifies molecular risk markers and clinical trajectories. Cancer Cell.

[28] Liu J, Lichtenberg T, Hoadley KA, Poisson LM, Lazar AJ, Cherniack AD (2018). An integrated TCGA Pan-Cancer Clinical data resource to drive high-quality survival outcome analytics. Cell.

[29] Mo Q, Wang S, Seshan VE, Olshen AB, Schultz N, Sander C (2013). Pattern discovery and cancer gene identification in integrated cancer genomic data. Proc Natl Acad Sci USA.

[30] Abida W, Cyrta J, Heller G, Prandi D, Armenia J, Coleman I (2019). Genomic correlates of clinical outcome in advanced prostate cancer. Proc Natl Acad Sci USA.

[31] Kumar A, Coleman I, Morrissey C, Zhang X, True LD, Gulati R (2016). Substantial interindividual and limited intraindividual genomic diversity among tumors from men with metastatic prostate cancer. Nat Med.

[32] Grasso CS, Wu YM, Robinson DR, Cao X, Dhanasekaran SM, Khan AP (2012). The mutational landscape of lethal castration-resistant prostate cancer. Nature.

[33] Chen K, Wu K, Jiao X, Wang L, Ju X, Wang M (2015). The endogenous cell-fate factor dachshund restrains prostate epithelial cell migration via repression of cytokine secretion via a cxcl signaling module. Cancer Res.

[34] Montanari M, Rossetti S, Cavaliere C, D’Aniello C, Malzone MG, Vanacore D (2017). Epithelial-mesenchymal transition in prostate cancer: an overview. Oncotarget.

[35] Wu K, Chen K, Wang C, Jiao X, Wang L, Zhou J (2014). Cell fate factor DACH1 represses YB-1-mediated oncogenic transcription and translation. Cancer Res.

[36] Safaei J, Manuch J, Gupta A, Stacho L, Pelech S (2011). Prediction of 492 human protein kinase substrate specificities. Proteome Sci.

[37] Hayakawa J, Mittal S, Wang Y, Korkmaz KS, Adamson E, English C (2004). Identification of promoters bound by c-Jun/ATF2 during rapid large-scale gene activation following genotoxic stress. Mol Cell.

[38] Wood CD, Thornton TM, Sabio G, Davis RA, Rincon M (2009). Nuclear localization of p38 MAPK in response to DNA damage. Int J Biol Sci.

[39] Picco V, Pages G (2013). Linking JNK activity to the DNA damage response. Genes Cancer.

[40] Lukas C, Savic V, Bekker-Jensen S, Doil C, Neumann B, Pedersen RS (2011). 53BP1 nuclear bodies form around DNA lesions generated by mitotic transmission of chromosomes under replication stress. Nat Cell Biol.

[41] Gunn A, Stark JM (2012). I-SceI-based assays to examine distinct repair outcomes of mammalian chromosomal double strand breaks. Methods Mol Biol.

[42] Jiao X, Velasco-Velazquez MA, Wang M, Li Z, Rui H, Peck AR (2018). CCR5 governs DNA damage repair and breast cancer stem cell expansion. Cancer Res.

[43] Sanchez-Vega F, Mina M, Armenia J, Chatila WK, Luna A, La KC (2018). Oncogenic signaling pathways in the cancer genome atlas. Cell.

[44] Di Vizio D, Sotgia F, Williams TM, Hassan GS, Capozza F, Frank PG (2007). Caveolin-1 is required for the upregulation of fatty acid synthase (FASN), a tumor promoter, during prostate cancer progression. Cancer Biol Ther.

[45] Trerotola M, Ganguly KK, Fazli L, Fedele C, Lu H, Dutta A (2015). Trop-2 is up-regulated in invasive prostate cancer and displaces FAK from focal contacts. Oncotarget.

[46] Di Vizio D, Morello M, Sotgia F, Pestell RG, Freeman MR, Lisanti MP (2009). An absence of stromal caveolin-1 is associated with advanced prostate cancer, metastatic disease and epithelial Akt activation. Cell Cycle.

[47] Williams TM, Hassan GS, Li J, Cohen AW, Medina F, Frank PG (2005). Caveolin-1 promotes tumor progression in an autochthonous mouse model of prostate cancer: genetic ablation of Cav-1 delays advanced prostate tumor development in tramp mice. J Biol Chem.

[48] Irshad S, Abate-Shen C (2013). Modeling prostate cancer in mice: something old, something new, something premalignant, something metastatic. Cancer Metastasis Rev.

[49] Kaplan-Lefko PJ, Chen TM, Ittmann MM, Barrios RJ, Ayala GE, Huss WJ (2003). Pathobiology of autochthonous prostate cancer in a pre-clinical transgenic mouse model. Prostate.

[50] Hurwitz AA, Foster BA, Allison JP, Greenberg NM, Kwon ED. The TRAMP mouse as a model for prostate cancer. Curr Protoc Immunol. 2001;Chapter 20: 20.5.1–20.5.23. 10.1002/0471142735.im2005s45 18432778

[51] Berman-Booty LD, Knudsen KE (2015). Models of neuroendocrine prostate cancer. Endocr Relat Cancer.

[52] Gelman IH (2016). How the TRAMP model revolutionized the study of prostate cancer progression. Cancer Res.

[53] Cerasuolo M, Maccarinelli F, Coltrini D, Mahmoud AM, Marolda V, Ghedini GC (2020). Modeling acquired resistance to the second-generation androgen receptor antagonist enzalutamide in the TRAMP model of prostate cancer. Cancer Res.

[54] Grabowska MM, DeGraff DJ, Yu X, Jin RJ, Chen Z, Borowsky AD (2014). Mouse models of prostate cancer: picking the best model for the question. Cancer Metastasis Rev.

[55] Augello MA, Liu D, Deonarine LD, Robinson BD, Huang D, Stelloo S (2019). CHD1 loss alters AR binding at lineage-specific enhancers and modulates distinct transcriptional programs to drive prostate tumorigenesis. Cancer Cell.

[56] Augello MA, Liu D, Deonarine LD, Robinson BD, Huang D, Stelloo S (2019). CHD1 loss alters AR binding at lineage-specific enhancers and modulates distinct transcriptional programs to drive prostate tumorigenesis. Cancer Cell.

[57] Baena E, Shao Z, Linn DE, Glass K, Hamblen MJ, Fujiwara Y (2013). ETV1 directs androgen metabolism and confers aggressive prostate cancer in targeted mice and patients. Genes Dev.

[58] Zarkowska T, Mittnacht S (1997). Differential phosphorylation of the retinoblastoma protein by G1/S cyclin-dependent kinases. J Biol Chem.

[59] Kong D, Liu Y, Liu Q, Han N, Zhang C, Pestell RG (2016). The retinal determination gene network: from developmental regulator to cancer therapeutic target. Oncotarget.

[60] Wu K, Yang Y, Wang C, Davoli MA, D’Amico M, Li A (2003). DACH1 inhibits transforming growth factor-beta signaling through binding Smad4. J Biol Chem.

[61] Jiao X, Li Z, Wang M, Katiyar S, Di Sante G, Farshchian M (2019). Dachshund depletion disrupts mammary gland development and diverts the composition of the mammary gland progenitor pool. Stem Cell Rep.

[62] Pal D, Pertot A, Shirole NH, Yao Z, Anaparthy N, Garvin T (2017). TGF-beta reduces DNA ds-break repair mechanisms to heighten genetic diversity and adaptability of CD44+/CD24- cancer cells. Elife.

[63] Zhang Q, Helfand BT, Jang TL, Zhu LJ, Chen L, Yang XJ (2009). Nuclear factor-kappaB-mediated transforming growth factor-beta-induced expression of vimentin is an independent predictor of biochemical recurrence after radical prostatectomy. Clin Cancer Res.

[64] Wikstrom P, Stattin P, Franck-Lissbrant I, Damber JE, Bergh A (1998). Transforming growth factor beta1 is associated with angiogenesis, metastasis, and poor clinical outcome in prostate cancer. Prostate.

[65] Ao M, Williams K, Bhowmick NA, Hayward SW (2006). Transforming growth factor-beta promotes invasion in tumorigenic but not in nontumorigenic human prostatic epithelial cells. Cancer Res.

[66] Walker L, Millena AC, Strong N, Khan SA (2013). Expression of TGFbeta3 and its effects on migratory and invasive behavior of prostate cancer cells: involvement of PI3-kinase/AKT signaling pathway. Clin Exp Metastasis.

[67] Bennardo N, Cheng A, Huang N, Stark JM (2008). Alternative-NHEJ is a mechanistically distinct pathway of mammalian chromosome break repair. PLoS Genet.

[68] Choi YE, Meghani K, Brault ME, Leclerc L, He YJ, Day TA (2016). Platinum and PARP Inhibitor Resistance Due to Overexpression of MicroRNA-622 in BRCA1-Mutant Ovarian Cancer. Cell Rep.

[69] Lee EK, Matulonis UA (2020). PARP inhibitor resistance mechanisms and implications for post-progression combination therapies. Cancers.

[70] Wang M, Wu W, Wu W, Rosidi B, Zhang L, Wang H (2006). PARP-1 and Ku compete for repair of DNA double strand breaks by distinct NHEJ pathways. Nucleic Acids Res.

[71] Isabelle M, Moreel X, Gagne JP, Rouleau M, Ethier C, Gagne P (2010). Investigation of PARP-1, PARP-2, and PARG interactomes by affinity-purification mass spectrometry. Proteome Sci.

[72] Paddock MN, Bauman AT, Higdon R, Kolker E, Takeda S, Scharenberg AM (2011). Competition between PARP-1 and Ku70 control the decision between high-fidelity and mutagenic DNA repair. DNA Repair.

[73] Lozano R, Castro E, Aragon IM, Cendon Y, Cattrini C, Lopez-Casas PP (2021). Genetic aberrations in DNA repair pathways: a cornerstone of precision oncology in prostate cancer. Br J Cancer.

[74] Gao J, Aksoy BA, Dogrusoz U, Dresdner G, Gross B, Sumer SO (2013). Integrative analysis of complex cancer genomics and clinical profiles using the cBioPortal. Sci Signal.

[75] Davis RJ, Shen W, Sandler YI, Amoui M, Purcell P, Maas R (2001). Dach1 mutant mice bear no gross abnormalities in eye, limb, and brain development and exhibit postnatal lethality. Mol Cell Biol.

[76] Pestell TG, Jiao X, Kumar M, Peck AR, Prisco M, Deng S (2017). Stromal cyclin D1 promotes heterotypic immune signaling and breast cancer growth. Oncotarget.

[77] Casimiro MC, Di Sante G, Ju X, Li Z, Chen K, Crosariol M (2016). Cyclin D1 promotes androgen-dependent DNA damage repair in prostate cancer cells. Cancer Res.

[78] Kravtsov O, Hartley CP, Comperat EM, Iczkowski KA (2019). KIF3B protein expression loss correlates with metastatic ability of prostate cancer. Am J Clin Exp Urol.

[79] Wu K, Katiyar S, Li A, Liu M, Ju X, Popov VM (2008). Dachshund inhibits oncogene-induced breast cancer cellular migration and invasion through suppression of interleukin-8. Proc Natl Acad Sci USA.

[80] Todaro GJ, Green H (1963). Quantitative studies of the growth of mouse embryo cells in culture and their development into established lines. J Cell Biol.

[81] Wu K, Liu M, Li A, Donninger H, Rao M, Jiao X (2007). Cell fate determination factor DACH1 inhibits c-Jun-induced contact-independent growth. Mol Biol Cell.

[82] Ai J, Pascal LE, Wei L, Zang Y, Zhou Y, Yu X (2017). EAF2 regulates DNA repair through Ku70/Ku80 in the prostate. Oncogene.

[83] Liu Q, Li A, Yu S, Qin S, Han N, Pestell RG (2018). DACH1 antagonizes CXCL8 to repress tumorigenesis of lung adenocarcinoma and improve prognosis. J Hematol Oncol.

[84] Li Z, Wang C, Jiao X, Lu Y, Fu M, Quong AA (2006). Cyclin D1 regulates cellular migration through the inhibition of thrombospondin 1 and ROCK signaling. Mol Cell Biol.

[85] Mayeur GL, Kung WJ, Martinez A, Izumiya C, Chen DJ, Kung HJ (2005). Ku is a novel transcriptional recycling coactivator of the androgen receptor in prostate cancer cells. J Biol Chem.

[86] Oliver MH, Harrison NK, Bishop JE, Cole PJ, Laurent GJ (1989). A rapid and convenient assay for counting cells cultured in microwell plates: application for assessment of growth factors. J Cell Sci.

[87] Li Z, Jiao X, Wang C, Shirley LA, Elsaleh H, Dahl O (2010). Alternative cyclin D1 splice forms differentially regulate the DNA damage response. Cancer Res.

[88] Gyori BM, Venkatachalam G, Thiagarajan PS, Hsu D, Clement MV. Corrigendum to OpenComet: an automated tool for comet assay image analysis [Redox Biol. Volume 2, 2014, Pages 457-465]. Redox Biol. 2021;40:101876. 10.1016/j.redox.2021.101876 PMC7897849 33558181

[89] Zhou J, Liu Y, Zhang W, Popov VM, Wang M, Pattabiraman N (2010). Transcription elongation regulator 1 is a co-integrator of the cell fate determination factor Dachshund homolog 1. J Biol Chem.

[90] Mermel CH, Schumacher SE, Hill B, Meyerson ML, Beroukhim R, Getz G (2011). GISTIC2.0 facilitates sensitive and confident localization of the targets of focal somatic copy-number alteration in human cancers. Genome Biol.

