# Supplementary material for: The DACH1 gene is frequently deleted in prostate cancer, restrains prostatic intraepithelial neoplasia, decreases DNA damage repair, and predicts therapy responses
Source: Oncogene. 2023 Apr 24;42(22):1857–73. doi: 10.1038/s41388-023-02668-9 (PMC10238272; doi:10.1038/s41388-023-02668-9)

Supplemental Figure 1

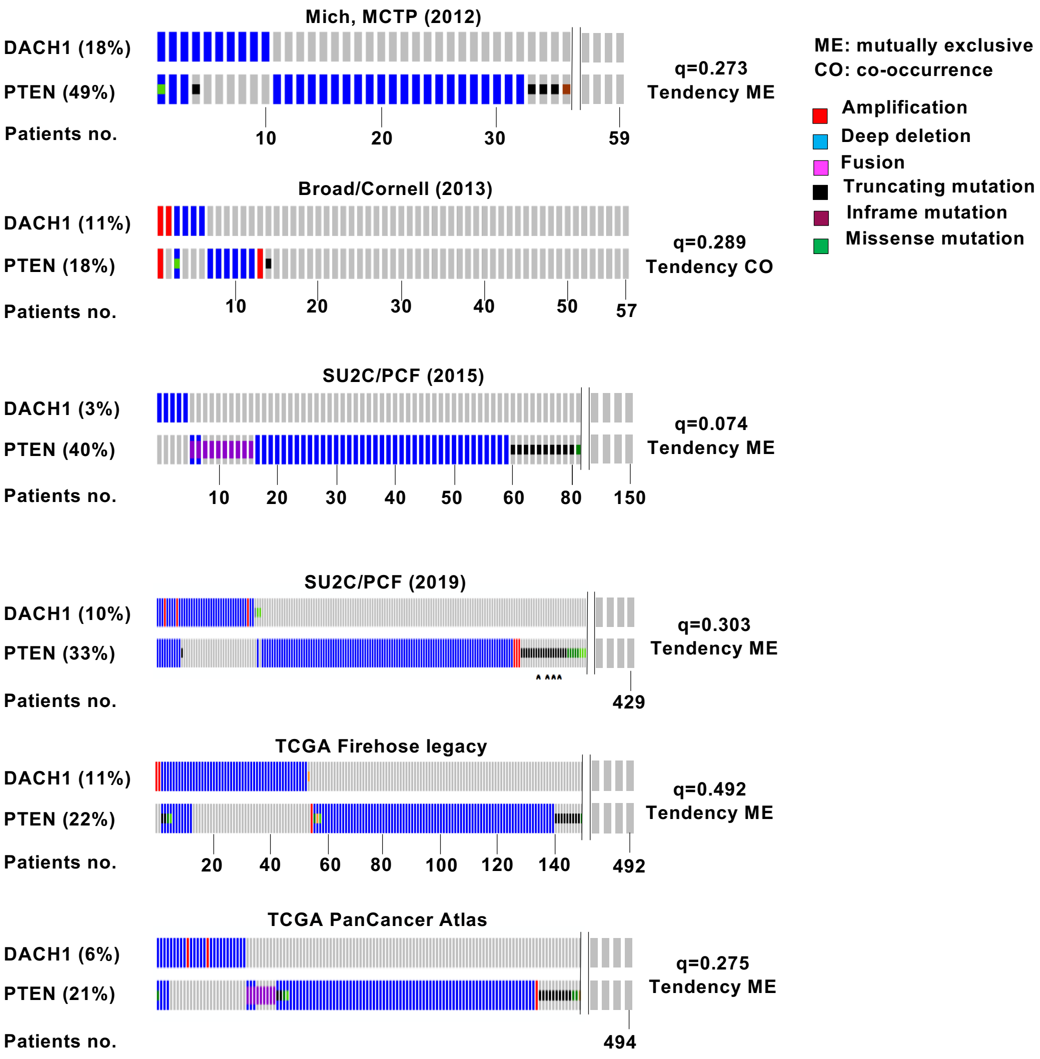

A

Mich, MCTP (2012)

DACH1 CNA

RB1 CNA	-2	-1	0	1	2	Sum
-2	9	4	1	0	0	14 Deep deletion
-1	1	15	8	0	0	24 Shallow deletion
0	1	2	19	0	0	22 Diploid
1	0	0	0	1	0	1 Gain
2	0	0	0	0	0	0 Amplified
Sum	11	21	28	1	0	61

Co-occurrence, q<0.001

B

Broad/Cornell (2013)

DACH1 CNA

RB1 CNA	-2	-1	0	1	2	NA	Sum
-2	3	0	2	0	0	0	5 Deep deletion
-1	1	13	1	0	0	0	15 Shallow deletion
0	0	1	33	0	1	0	35 Diploid
1	0	0	0	0	0	0	0 Gain
2	0	0	0	0	1	0	1 Amplified
NA	0	0	0	0	0	1	1 NA
Su	4	14	36	0	2	1	57

Co-occurrence, q<0.001

C

SU2C/PCF (2015)

DACH1 CNA

RB1 CNA	-2	-1	0	1	2	Sum
-2	0	6	2	2	0	10 Deep deletion
-1	0	3	0	0	0	3 Shallow deletion
0	5	74	51	7	0	137 Diploid
1	0	0	0	0	0	0 Gain
2	0	0	0	0	0	0 Amplified
Sum	5	83	53	9		150

Mutual exclusivity, q=0.63

D

SU2C/PCF (2019)

DACH1 CNA

RB1 CNA	-2	-1	0	1	2	Sum
-2	6	19	12	4	2	43 Deep deletion
-1	21	153	46	5	0	225 Shallow deletion
0	9	16	114	5	0	144 Diploid
1	1	8	14	8	1	32 Gain
2	0	0	0	0	0	0 Amplified
Sum	37	196	186	22	3	444

Mutual exclusivity, q=0.013

E

TCGA Firehose legacy

DACH1 CNA

RB1 CNA	-2	-1	0	1	2	Sum
-2	44	3	32	2	0	81 Deep deletion
-1	3	113	22	0	1	139 Shallow deletion
0	4	9	255	0	1	269 Diploid
1	0	1	0	2	0	3 Gain
2	0	0	0	0	0	0 Amplified
Sum	51	126	309	4	2	492

Co-occurrence, q<0.001

Supplemental Figure 3

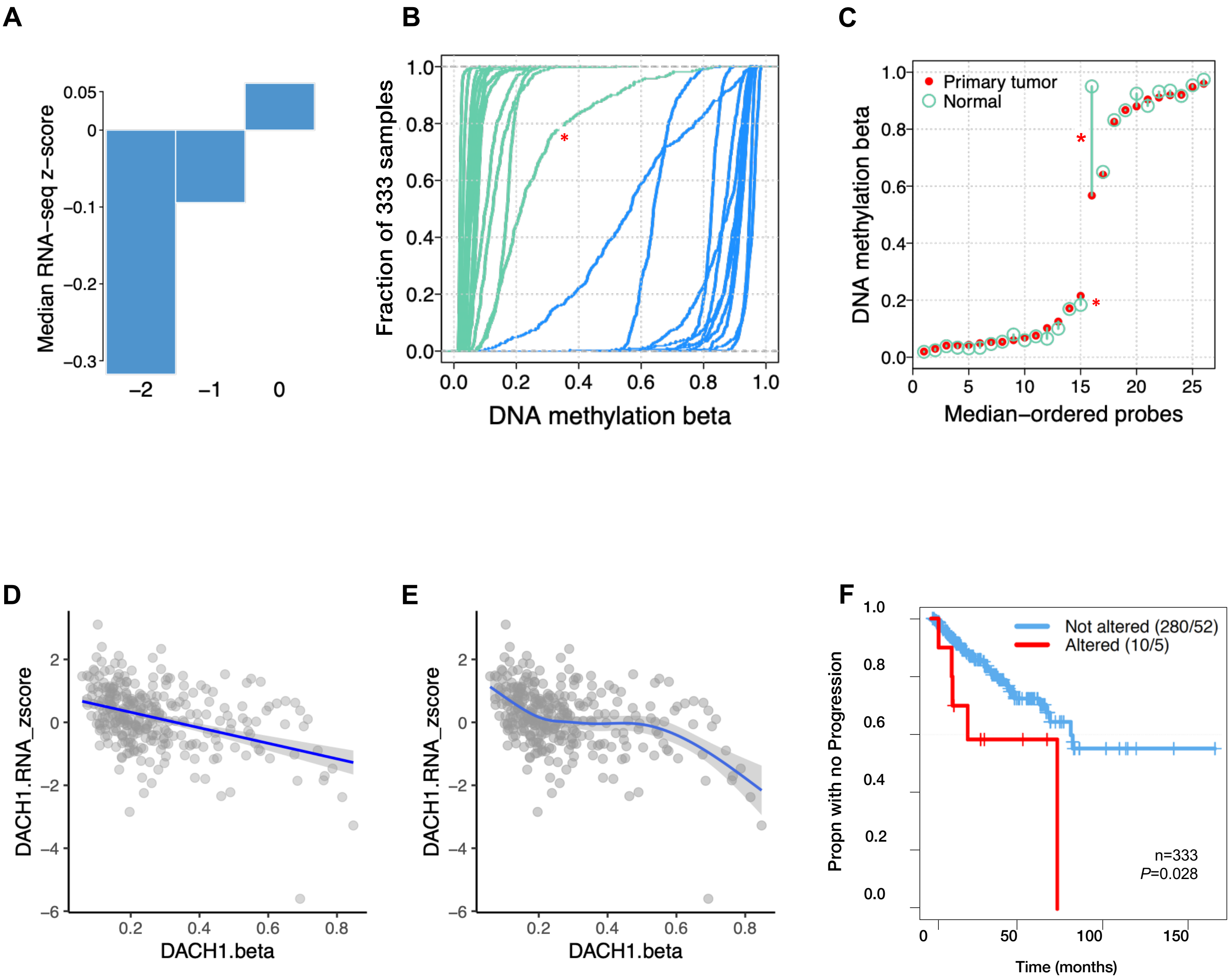

Supplemental Figure 4

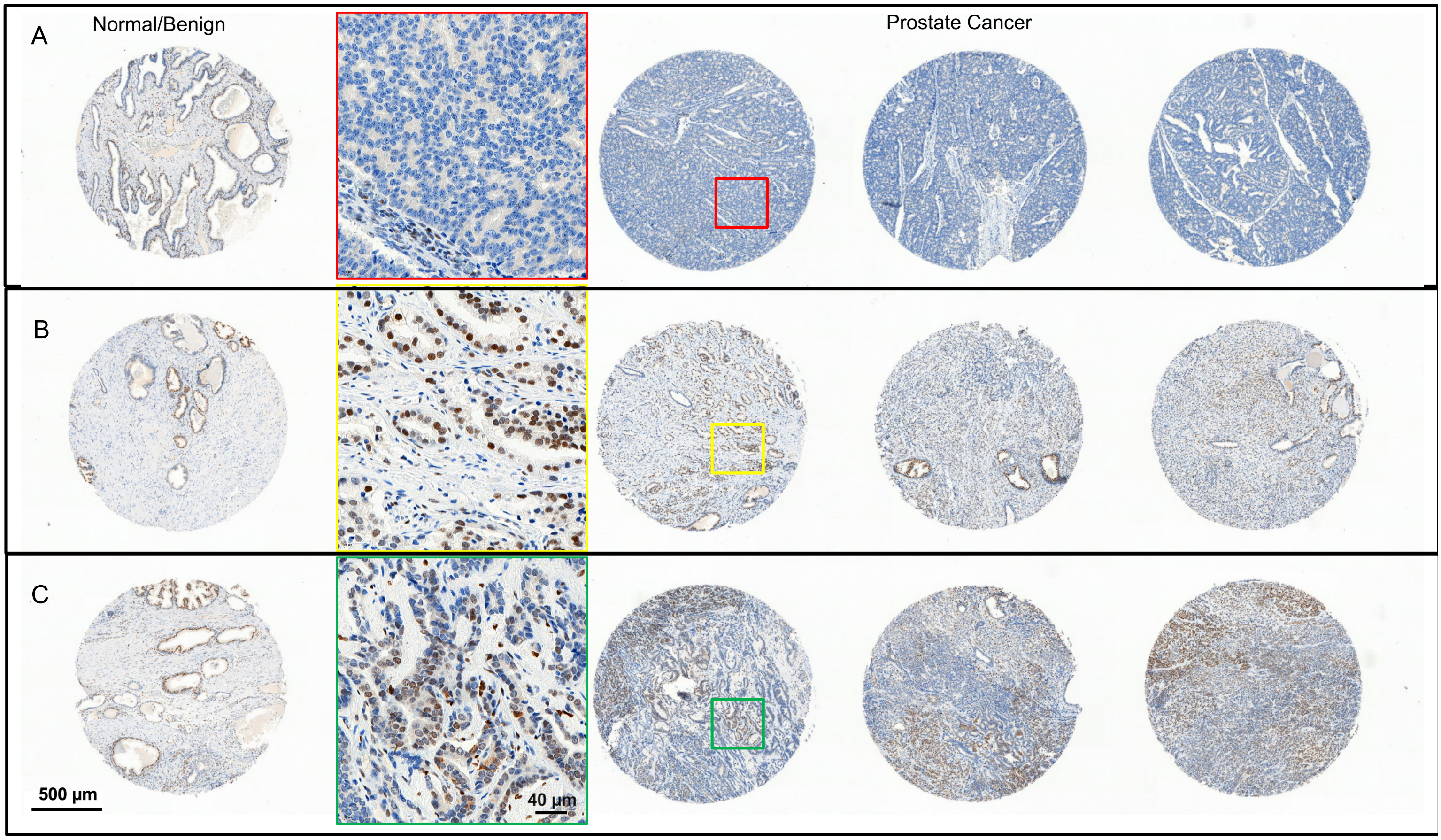

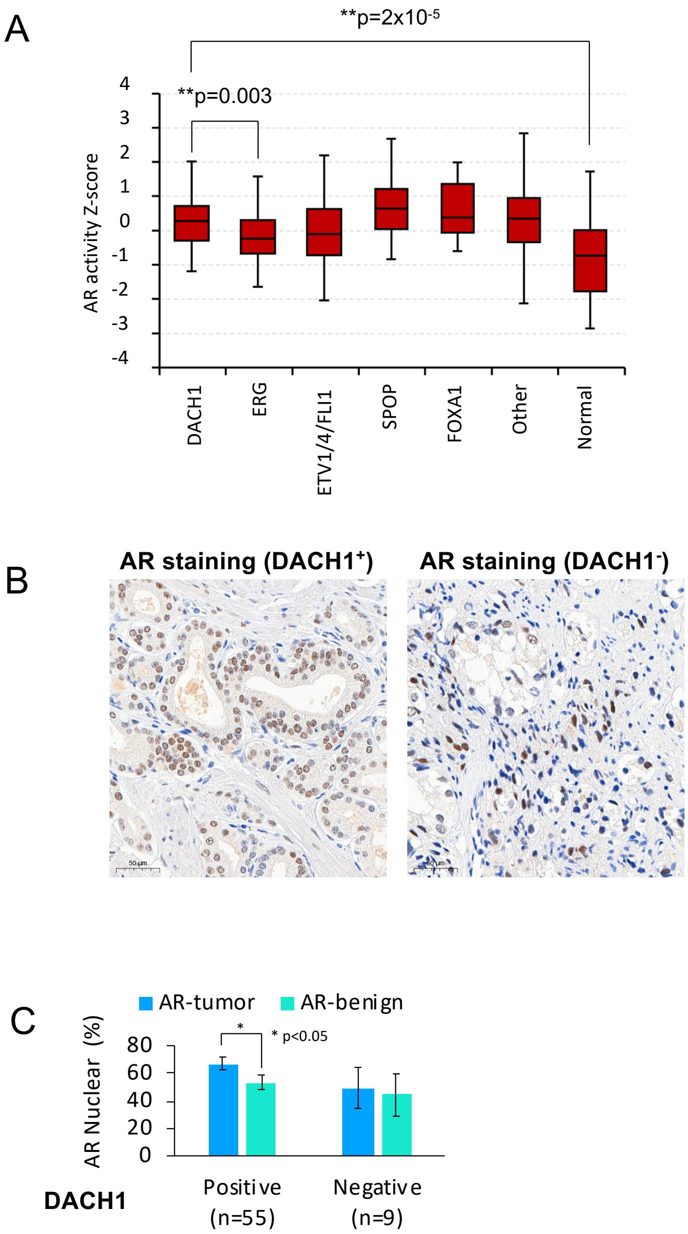
A
**p=2x10-5
**p=0.003
AR activity Z-score
4
3
2
1
0
-1
-2
-3
-4
DACH1
ERG
ETV1/4/FLI1
SPOP
FOXA1
Other
Normal
B
AR staining (DACH1+)
AR staining (DACH1-)
C
AR-tumor
AR-benign
AR Nuclear (%)
80
60
40
20
0
*
* p<0.05
DACH1
Positive
(n=55)
Negative
(n=9)

Supplemental Figure 6

A

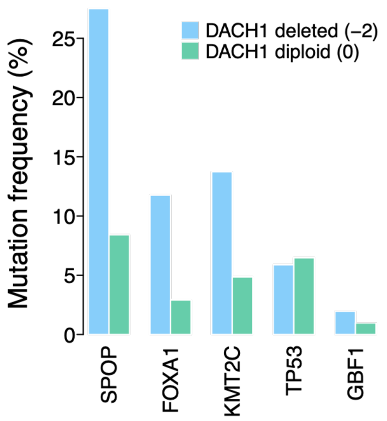

DACH1 SCNA	-2	-1	0	1	2	Sum
Samples	51	126	309	4	2	492
	Del		Dip			

	DACH1 Del (n=51)	DACH1 diploid (n=309)	p val Fisher test	p.adj (Bonf)
SPOP	14	26	0.0023	0.012
FOXA1	6	9	0.0158	0.079
KMT2C	7	15	0.0583	0.29
TP53	3	20	1.0	1.0
GBF1	1	3	0.4617	1.0

B

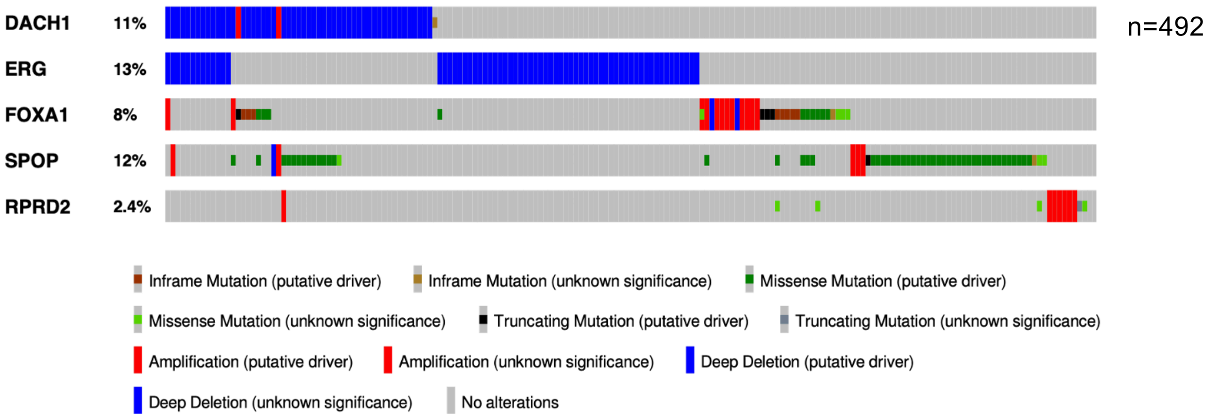

C

A	B	Neither	A Not B	B Not A	Both	Log2 Odds Ratio	p-Value	q-Value	Tendency
DACH1	ERG	386	41	52	13	1.235	0.015	0.051	Co-occurrence
	FOXA1	407	45	31	9	1.393	0.022	0.054	Co-occurrence
	SPOP	394	37	44	17	2.041	<0.001	<0.001	Co-occurrence
	RPRD2	427	53	11	1	-0.449	0.613	0.613	Mutual exclusivity
ERG	FOXA1	389	63	38	2	-1.622	0.078	0.156	Mutual exclusivity
	SPOP	367	64	60	1	<-3	0.001	0.006	Mutual exclusivity
	RPRD2	415	65	12	0	<-3	0.179	0.256	Mutual exclusivity
FOXA1	SPOP	398	33	54	7	0.645	0.214	0.267	Co-occurrence
	RPRD2	442	38	10	2	1.218	0.254	0.282	Co-occurrence
SPOP	RPRD2	422	58	9	3	1.278	0.177	0.256	Co-occurrence

D

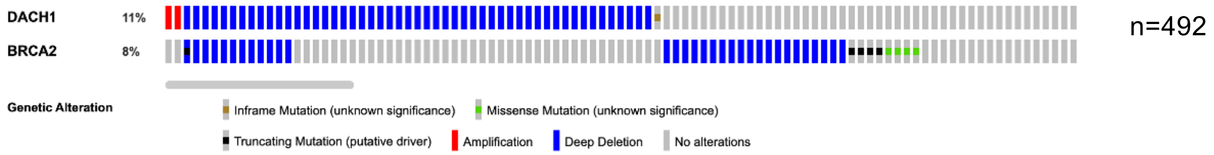

A	B	Neither	A Not B	B Not A	Both	Log2 Odds Ratio	p-Value	q-Value ▲	Tendency
DACH1	BRCA2	410	42	28	12	2.065	<0.001	**0.007**	**Co-occurrence**

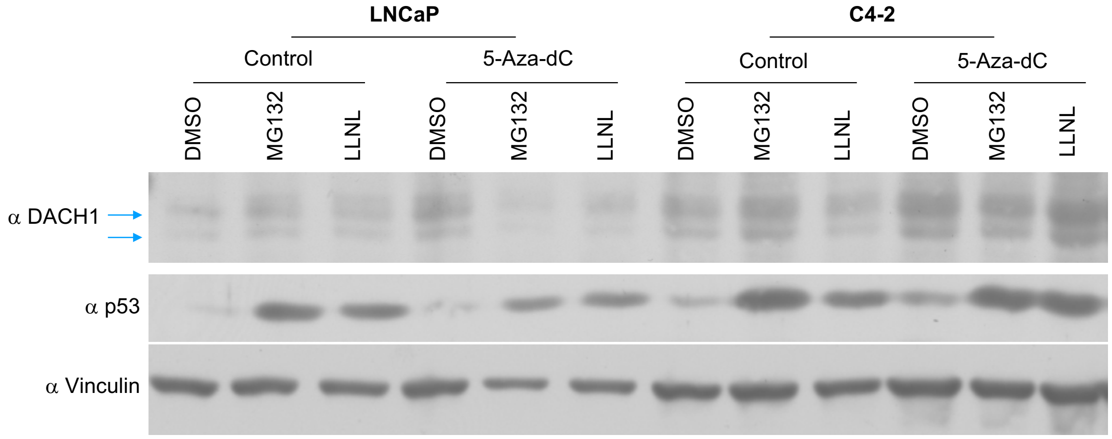
LNCaP
C4-2
Control
5-Aza-dC
Control
5-Aza-dC
DMSO
MG132
LLNL
DMSO
MG132
LLNL
DMSO
MG132
LLNL
DMSO
MG132
LLNL
α DACH1
α p53
α Vinculin

A

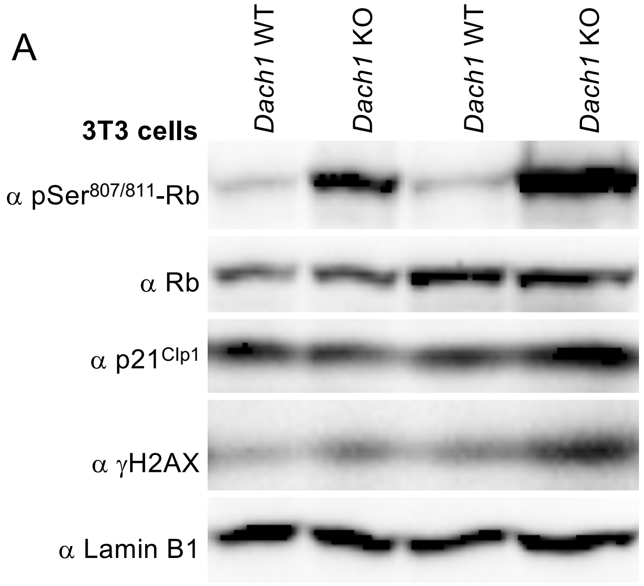
3T3 cells
Dach1 WT
Dach1 KO
Dach1 WT
Dach1 KO
α pSer807/811-Rb
α Rb
α p21Cip1
α γH2AX
α Lamin B1

B

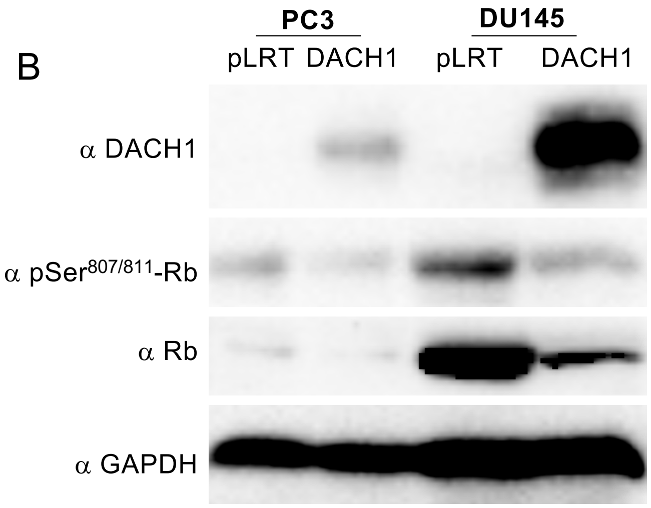
PC3
DU145
pLRT
DACH1
pLRT
DACH1
α DACH1
α pSer807/811-Rb
α Rb
α GAPDH

Supplemental Figure 9

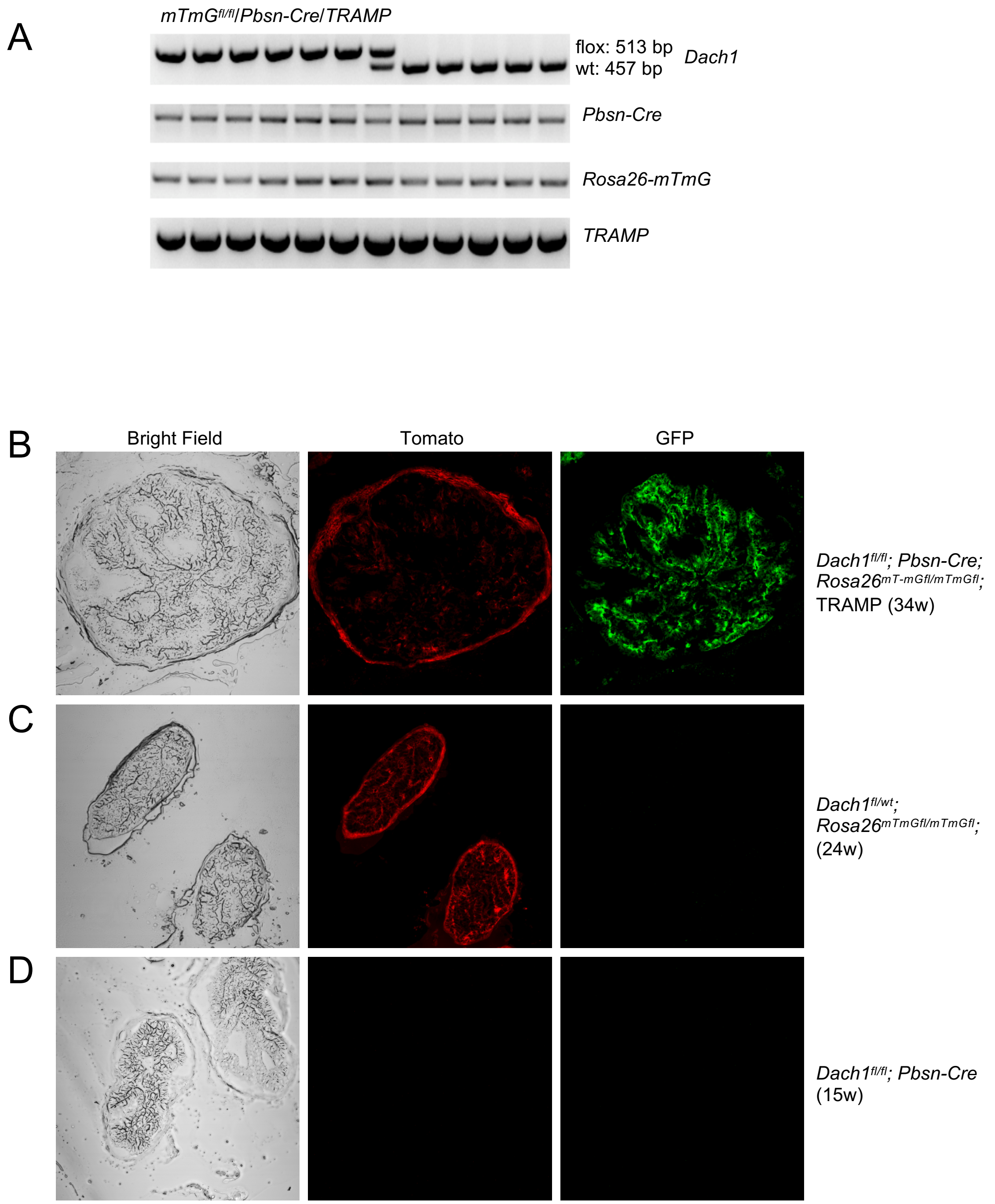

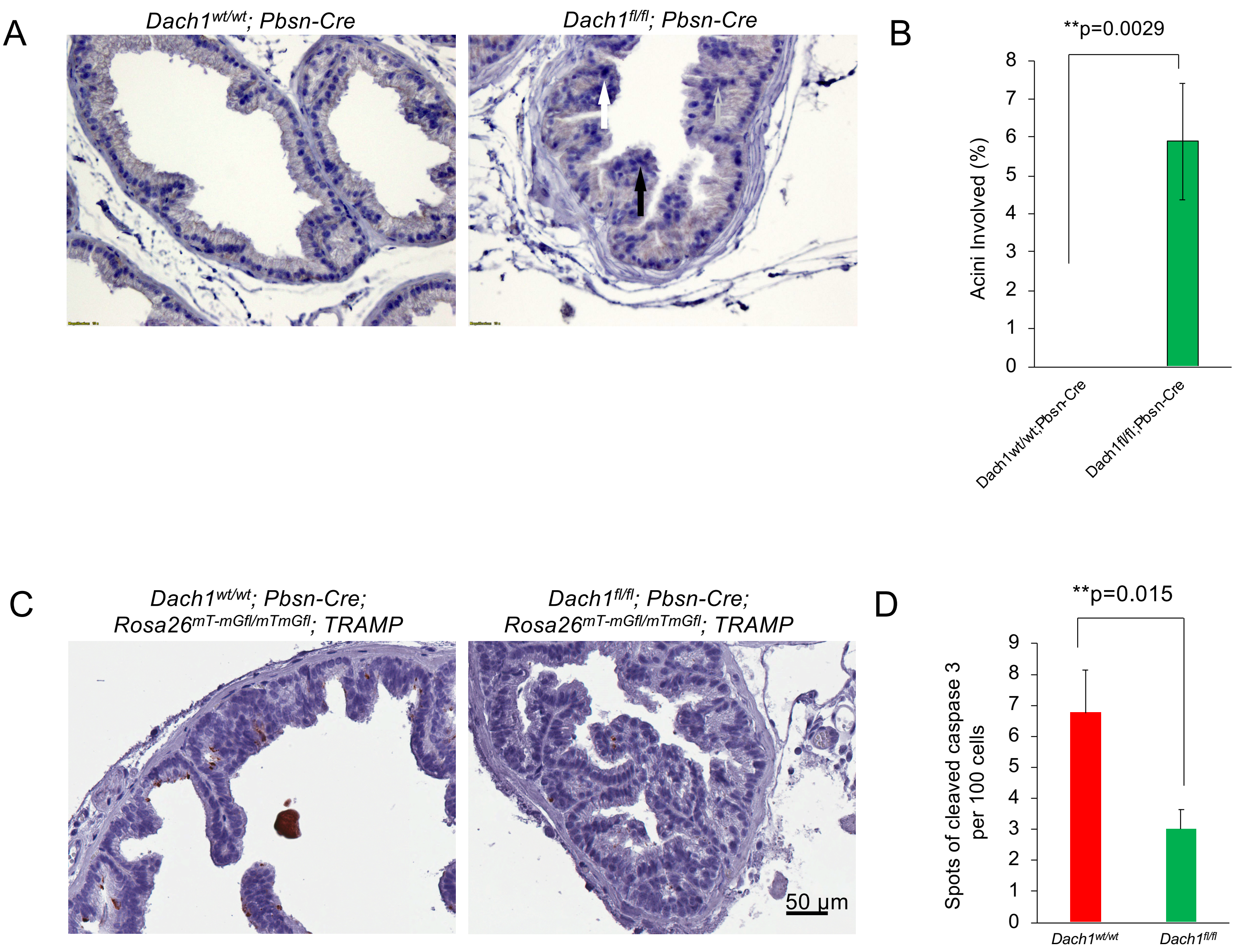
A
Dach1wt/wt; Pbsn-Cre
Dach1fl/fl; Pbsn-Cre
B
**p=0.0029
Acini Involved (%)
0
1
2
3
4
5
6
7
8
Dach1wt/wt;Pbsn-Cre
Dach1fl/fl;Pbsn-Cre
C
Dach1wt/wt; Pbsn-Cre; Rosa26mT-mGfl/mTmGfl; TRAMP
Dach1fl/fl; Pbsn-Cre; Rosa26mT-mGfl/mTmGfl; TRAMP
50 µm
D
**p=0.015
Spots of cleaved caspase 3 per 100 cells
0
1
2
3
4
5
6
7
8
9
Dach1wt/wt
Dach1fl/fl

A

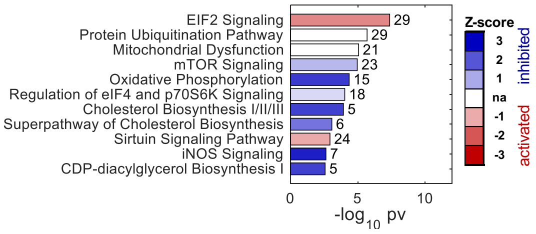

B

**Canonical Pathway**	***p***	**WT/KO Z**	**N**
EIF2 Signaling	4.27E-08	1.41	29
Protein Ubiquitination Pathway	2.04E-06	NaN	29
Mitochondrial Dysfunction	8.71E-06	NaN	21
mTOR Signaling	1.1E-05	-0.83	23
Oxidative Phosphorylation	4.37E-05	-2.32	15
Regulation of eIF4 and p70S6K Signaling	9.33E-05	-0.45	18
Cholesterol Biosynthesis I	0.000117	-2.24	5
Cholesterol Biosynthesis II (via 24,25-dihydrolanosterol)	0.000117	-2.24	5
Cholesterol Biosynthesis III (via Desmosterol)	0.000117	-2.24	5
Superpathway of Cholesterol Biosynthesis	0.000851	-1.63	6
Sirtuin Signaling Pathway	0.001175	1	24
iNOS Signaling	0.002344	-2.65	7
CDP-diacylglycerol Biosynthesis I	0.002692	-2.24	5

Supplemental Figure 12

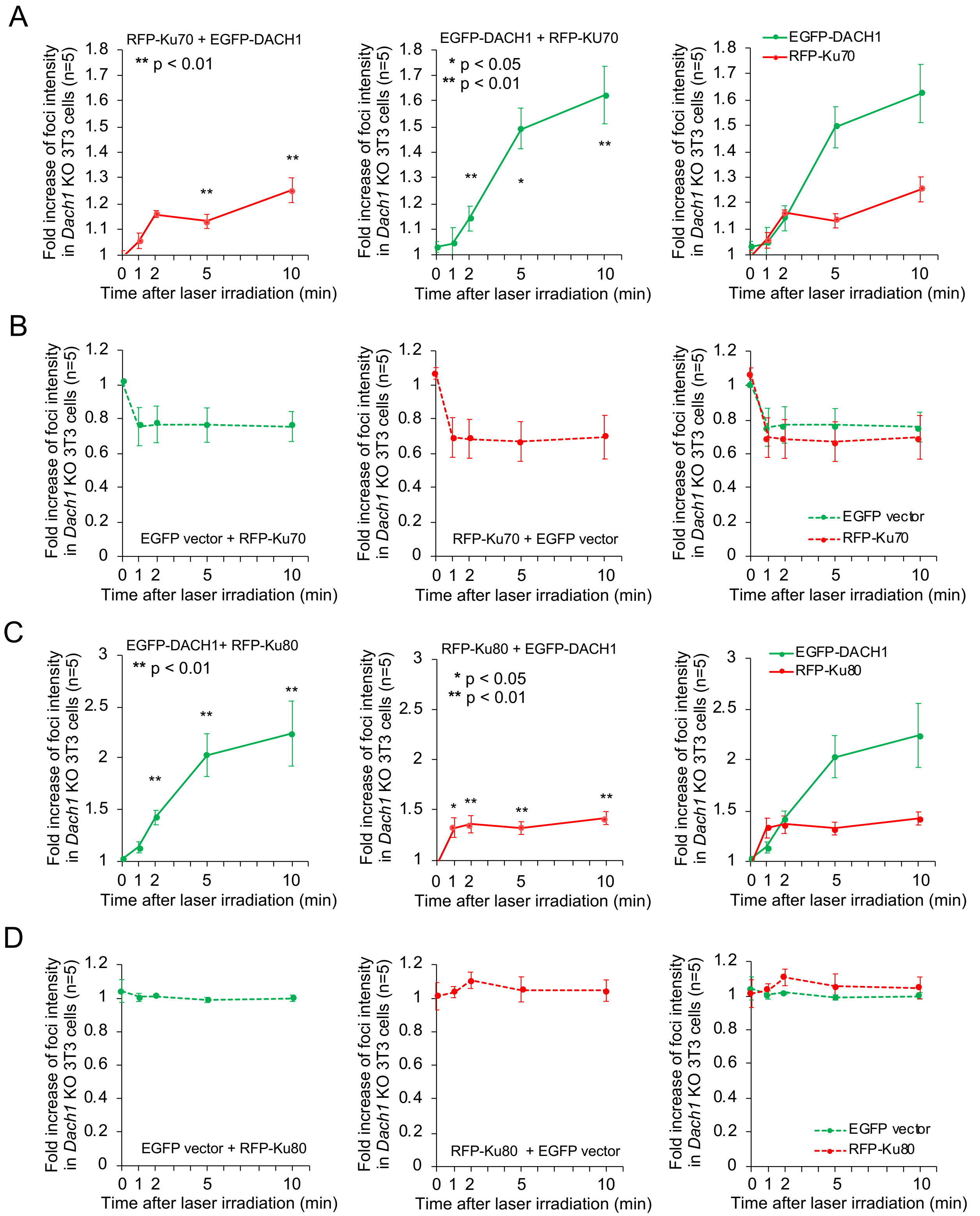

Supplemental Figure 13

A

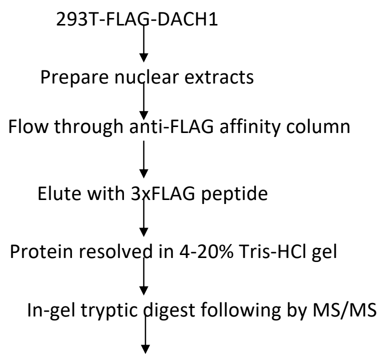

11	C2	ATP-dependent DNA helicase 2 subunit 2 (EC 3.6.1.-) (ATP-dependent DNA helicase II 80 kDa subunit)
15	G2	ATP-dependent DNA helicase 2 subunit 1 (ATP-dependent DNA helicase II 70 kDa subunit) (Lupus Ku aut)

B

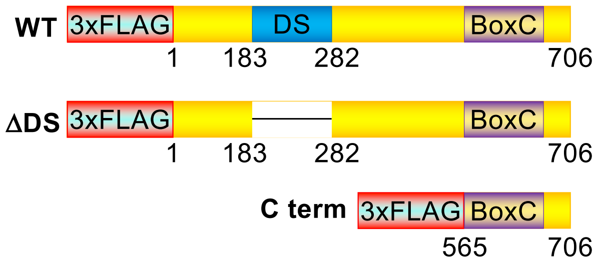

C

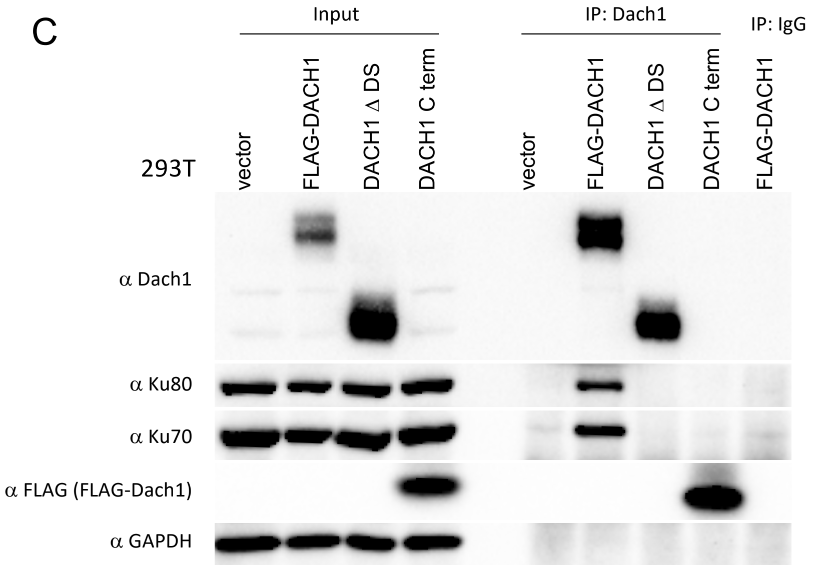

D

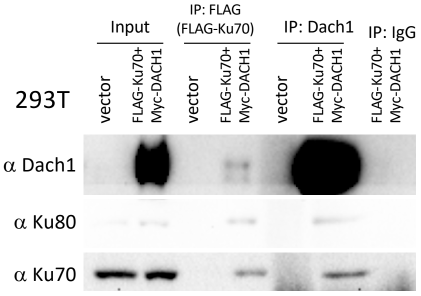

E

Kinase Predictor V2 Score	Human Kinase Short Name	Human UniProt. ID	Kinase Predictor V2 Score - Proximity
639	ERK1	P27361	1440
629	ERK2 (MAPK1)	P28482	1439
596	JNK1 (MAPK8)	P45983	1367
596	JNK3 (MAPK10)	P53779	1367
594	ERK5 (MAPK7)	Q13164	1347
590	p38g MAPK (MAPK1	P53778	1337
580	CDK2	P24941	1327
578	p38a MAPK (MAPK1	Q16539	1326
578	CDK1 (CDC2)	P06493	1319
575	JNK2 (MAPK9)	P45984	1316
575	p38d MAPK (MAPK1	O15264	1313
561	CDKL5 (STK9)	O76039	1299
557	CDK3	Q00526	1284
552	p38b MAPK (MAPK1	Q15759	1278

Supplemental Figure 14

A

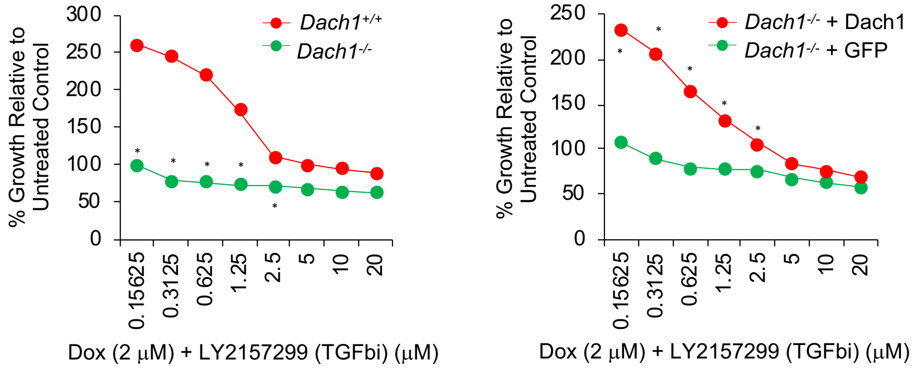

B

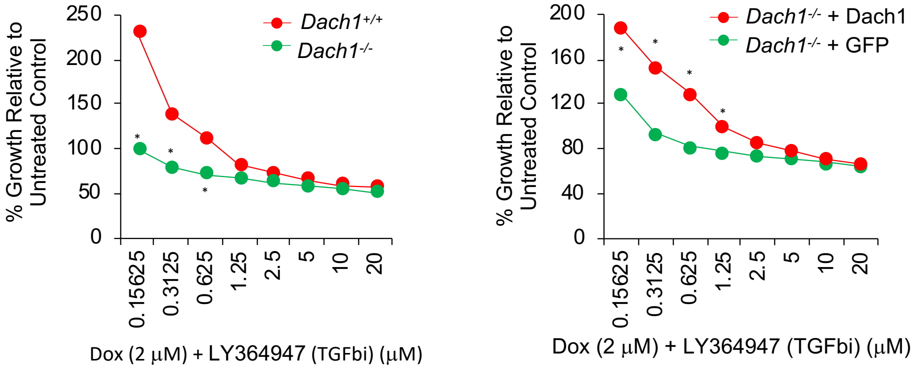

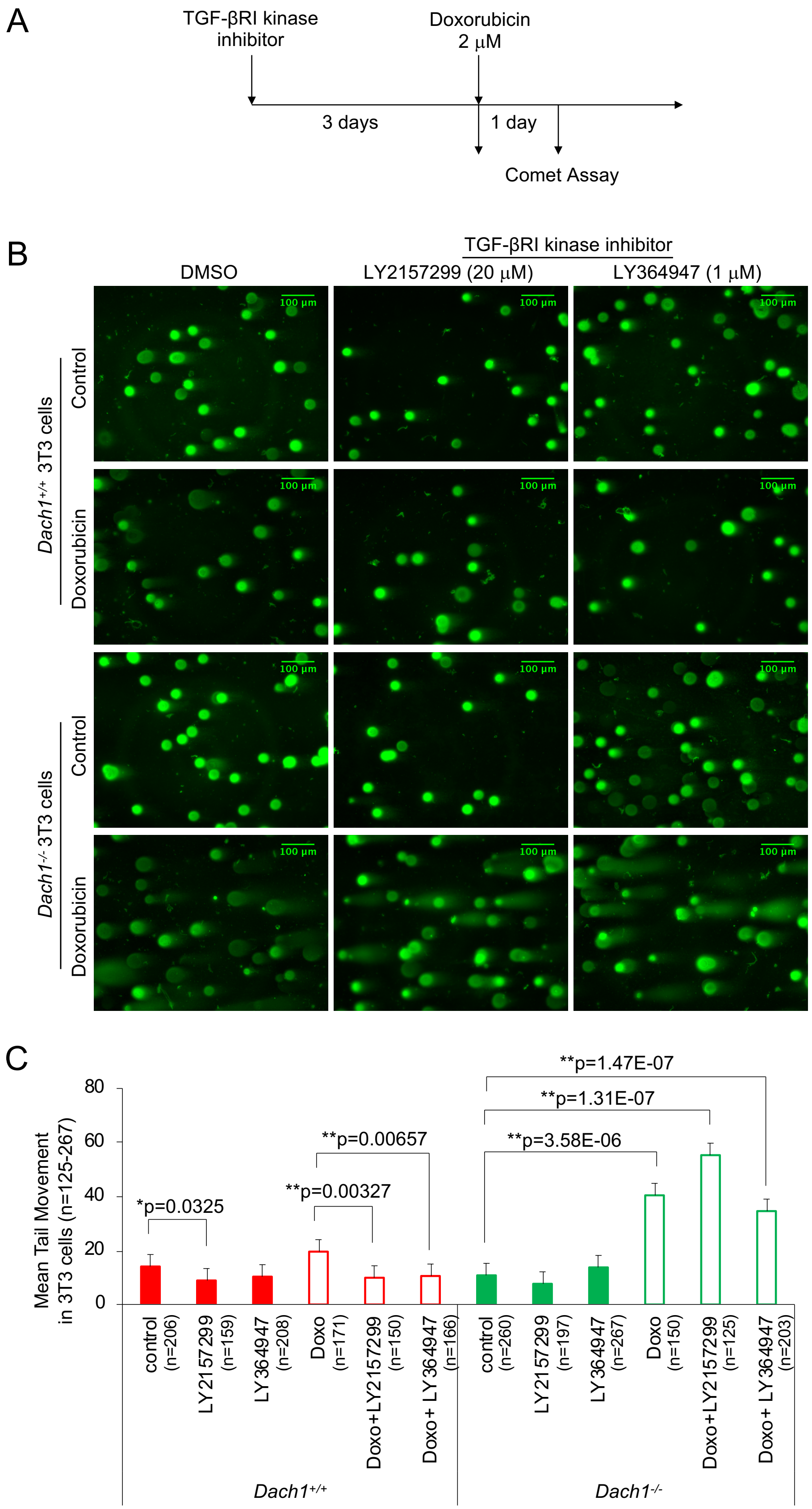
A
TGF-βRI kinase inhibitor
Doxorubicin 2 μM
3 days
1 day
Comet Assay
B
TGF-βRI kinase inhibitor
DMSO
LY2157299 (20 μM)
LY364947 (1 μM)
Dach1+/+ 3T3 cells
Dach1-/- 3T3 cells
Control
Doxorubicin
100 μm
C
Mean Tail Movement in 3T3 cells (n=125-267)
*p=0.0325
**p=0.00327
**p=0.00657
**p=3.58E-06
**p=1.31E-07
**p=1.47E-07
control (n=206)
LY2157299 (n=159)
LY364947 (n=208)
Doxo (n=171)
Doxo+LY2157299 (n=150)
Doxo+ LY364947 (n=166)
control (n=260)
LY2157299 (n=197)
LY364947 (n=267)
Doxo (n=150)
Doxo+LY2157299 (n=125)
Doxo+ LY364947 (n=203)
Dach1+/+
Dach1-/-

Supplemental Figure 16

A

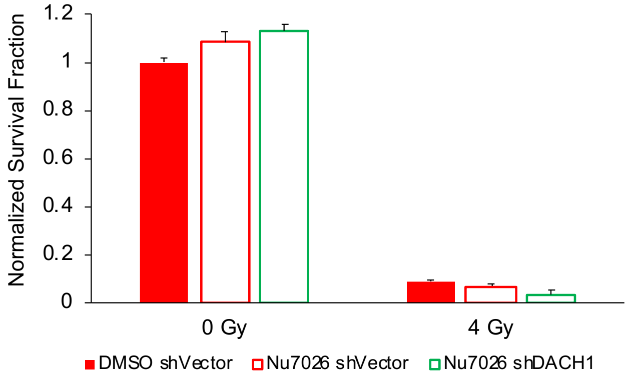

B

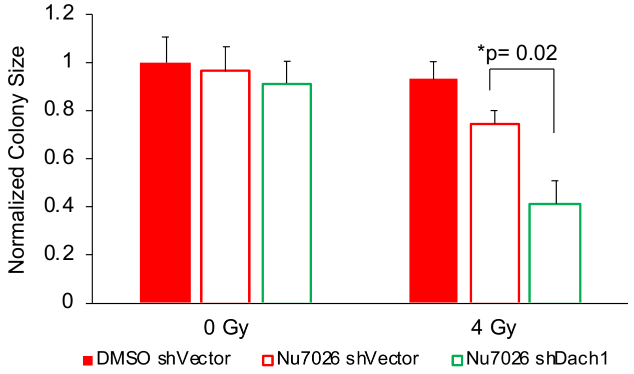

Supplement: Supplementary file 1 — Supplemental Figures [file 41388_2023_2668_MOESM1_ESM.pdf]
