# Supplementary material for: The DACH1 gene is frequently deleted in prostate cancer, restrains prostatic intraepithelial neoplasia, decreases DNA damage repair, and predicts therapy responses
Source: Oncogene. 2023 Apr 24;42(22):1857–73. doi: 10.1038/s41388-023-02668-9 (PMC10238272; doi:10.1038/s41388-023-02668-9)
Supplement: Supplementary file 2 — Supplemental Figure Legends [file 41388_2023_2668_MOESM2_ESM.docx]

**The *DACH1* gene is frequently deleted in prostate cancer, restrains prostatic intraepithelial neoplasia, augments DNA damage repair and predicts therapy responses.**

**Supplemental Figure 1. The *DACH1* gene is rarely co-deleted with *PTEN* in human prostate cancer**. For six cohorts, we used cBioPortal to generate default oncoprints for DACH1 and PTEN, showing copy number alterations, mutations, and fusions. Alignment of *DACH1* gene deletion and *PTEN* deletion in the same patients shows deletion of each gene shows a trend towards mutually exclusivity in 5 of the data sets.

**Supplemental Figure 2. Copy number alterations (CNA) for *DACH1* and *RB1*.** Thresholded CNA data from cBioPortal for these two genes we downloaded, for a number of cohorts. The tables were calculated with a custom R script.

**Supplemental Figure 3. DACH1 expression and DNA methylation.** (A). Relationship between median DACH1 RNA-Seq z-scores and somatic copy number variation, from cBioPortal data for the TCGA Firehose Legacy Prostate Adenocarcinoma cohort, for N=491 primary tumor samples. (B). DACH1 mRNA expression *vs.* DACH1 DNA methylation. Empirical (cumulative) distribution functions (EDFs) for DNA methylation beta values for 502 primary tumor samples (PanCancer Atlas). Fifteen of 26 Illumina 450K probes with data for both primary tumors and adjacent tissue normals were within approximately 2 kb of the DACH1 transcription start site (TSS) (green EDFs *vs*. blue EDFs). The EDF with the red asterisk * shows the EDF for the DACH1 probe reported by cBioPortal: cg13726218. (C). Median per-probe beta values for n=502 primary tumors (red dots) and 50 adjacent tissue normals (green circles), ordered by median tumor beta values. The red asterisk * shows the DACH1 probe reported by cBioPortal: cg13726218. (D,E). For DACH1-diploid samples (N=308), the relationship between the probe cg13726218 DACH1 DNA methylation beta and DACH1 RNA-Seq expression z-score is shown as (D) a linear regression best-fit line and (E) a nonlinear regression Generalized Additive Model (GAM) best-fit line. (F). DACH1 gene expression, defined by Z score, correlates with outcomes ([[1](#_ENREF_1)], n=290 of 333 samples). We obtained RSEM expression for DACH1 and defined “altered DACH1” samples as those in which DACH1’s RSEM Z-score was below -2.0. Using the PanCancer outcomes [[2](#_ENREF_2)], segregating the samples by low RSEM returned a Kaplan-Meier log-rank *P* value of 0.028 for Progression Free Interval (PFI) outcome data based on biochemical recurrence (BCR) as shown.

**Supplemental Figure 4**. Immunohistochemistry for DACH1 protein shown for three representative cases (***a-c***) of prostate cancer with matched nearby normal/benign tissue. DACH1 is consistently expressed in normal/benign prostate epithelial cells, but lost in prostate cancer cells of Gleason grade 5 cancer in Case a, and retained in Cases b with Gleason grade 3 cancer and Case c with Gleason grade 4 cancer. Note positive DACH1 staining in some stromal cells (lower left corner of high power field) of Case a. There was a grade-wise decrease in staining.

**Supplemental Figure 5. *DACH1* deletion PCa are enriched for AR activity.** (A). Boxplots of AR activity z-scores are shown for different subtypes of human PCa in Figure 2A. (B). Immunohistochemistry for AR protein on DACH1^+^ and DACH1^-^ human prostate cancer samples in Supplemental Figure 4. (C). Quantification of the % AR nuclear staining by IHC on DACH1^+^ (n=55) vs. DACH1^-^ (n=9) prostate cancer samples.

**Supplemental Figure 6. PCa with *DACH1* deep deletions are enriched for *BRCA2* mutations.** (A). Mutation frequencies of five known PCa oncogenes or tumor suppressors, comparing primary tumor samples with deeply deleted DACH1 (n=51) *vs*. diploid DACH1 (n=309), from cBioPortal PCa data for the TCGA Firehose Legacy cohort (n=492). For the Firehose Legacy cohort at cBioPortal (n=492 primary tumors), we downloaded thresholded copy number alterations (CNA), mutations, and RSEM normalized gene expression data for *DACH1, ERG, ETV1, FOXA1, GBF1, KMT2C, SPOP, TP53*, and *RPRD2*. For n=360 records with deep homozygous *DACH1* deletions (i.e. GISTIC CNA=-2, n=51), and diploid *DACH1* (i.e. GISTIC CNA=0, n=309), we calculated the percent of mutated samples for *SPOP, FOXA1, KMT2C*, *TP53* and *GBF1*. We excluded ERG because, in this cohort, it had no mutations, and excluded ETV1 because it had only 1 mutation. We displayed the results as barplots. For the five genes that we retained, we used a Fisher exact test on count data to calculate p values for the mutation frequencies of DACH1-deleted *vs.* DACH1 diploid samples, and Bonferroni-corrected the p values for multiple hypothesis testing by multiplying them by five. (B). cBioPortal oncoprint for copy number alterations and mutations in *DACH1, ERG, FOXA1, SPOP* and *RPRD2*, with (C). a table of mutual exclusivity and co-occurrence patterns for gene pairs, for this cohort. For the five retained genes in (B), *DACH1, ERG, FOXA1, SPOP* and *RPRD2*, we generated a default cBioPortal oncoprint and reported cBioPortal mutual exclusivity and co-occurrence tendencies for all pairs of genes (C). (D). cBioPortal oncoprint for copy number alterations and mutations in *DACH1* and *BRCA2,* with a table of co-occurrence patterns for *DACH1* deletions and *BRCA2* mutations.

**Supplemental Figure 7. DACH1 expression is restrained by DNA methylation.** LNCaP and C4-2 cells were treated with the DNA methylase inhibitor 5-Aza-dC (10 μM, with either control, the 26S proteasome inhibitors MG132 (20 μM), or N-acetyl-L-leucyl-L-leucyl-L-nor leucinal (LLNL) (25 μM). Western blot was conducted for the proteins indicated (DACH1, p53) and the protein loading control vinculin.

**Supplemental Figure 8. Genetic deletion of *Dach1* does not reduce pRB.** (A). Western blot analysis conducted using total cellular protein lysates from *Dach1*^+/+^ and *Dach1*^-/-^ 3T3 cells. with the antibodies as shown in the figure (rabbit polyclonal anti-Phospho-Rb (Ser807/811) (CST#9308), mouse monoclonal anti-Rb (Oncogene Science #OP28), rabbit polyclonal anti-p21^Cip1^ (sc-397), mouse monoclonal anti-Phospho-Histone H2A.X (Ser139) (CST#80312), and the loading control Lamin B1 rabbit polyclonal antibody (ab16048). (B). Western blot analysis of total cellular protein lysates from PC3 and DU145 cells stably expressing a tetracycline inducible DACH1 or pLRT vector control. Cells were treated with 1 μg/ml doxycycline for 2 days. Antibodies were used as shown in the figure (rabbit polyclonal anti-DACH1 (Proteintech#10914-1-AP), rabbit polyclonal anti-Phospho-Rb (Ser807/811) (CST#9308), mouse monoclonal anti-Rb (Oncogene Science #OP28), and the loading control GAPDH rabbit polyclonal antibody (sc-25778)).

**Supplemental Figure 9. Genetic characterizaion of multigenic mice.** (A). Representative example of GEMM genotyping (mouse tail DNA) by PCR showing detection of the probasin-Cre (Pbsn-Cre), ROSA26^mT/mG^, and TRAMP transgenes. The floxed *Dach1* allele is detected by the presence of 513 bp *Dach1* floxed allele by PCR. (B). Representative example of a transgenic mouse prostate showing phase contrast bright field (BF), immunofluorescence for tomato red fluorescent protein (mT) and green fluorescent protein (mG). Prostate specific Cre expression (Pbsn-Cre) converts constitutive red fluorescent protein into green fluorescent protein (mG). (C). Analysis of prostate from transgenic mice in which red fluorescence is found throughout the prostate in the absence of Pbsn-Cre expression. (D). Prostate from transgenic mice that do not express the ROSA26^mT/mG^ transgene.

**Supplemental Figure 10. Histological analysis of prostate specific *Dach1* deletion mice.**

(A,B). Representative histology of Probasin-Cre-Dach1^wt/wt^ *vs.* Probasin-Cre- Dach1^fl/fl^ mice. Features of prostatic intraepithelial neoplasia (PIN) in the Probasin-Cre- Dach1^fl/fl^ mice include increased cellular proliferation of ductal columnar epithelium creating multiple layers of cells within the duct, loss of cellular polarity (black arrow), nuclear enlargement (white arrow) presence of prominent nucleoli (gray arrow). Data are representative of 5 mice, three Probasin-Cre- *Dach1^wt/wt^* mice and two Probasin-Cre- *Dach1^fl/fl^* mice. Two slides of HE staining were viewed for each mice. Data are shown as mean ± SEM for percentage of acini involved in focal low grade PIN (N=6 for Probasin-Cre- *Dach1^wt/wt^* mice, N=4 for Probasin-Cre- *Dach1^fl/fl^* mice). (C,D). Representative immunohistochemistry with results shown as mean ± SEM for Cleaved Caspase-3 in mouse prostates of Probasin-Cre-*Dach1^fl/fl^* ROSA26^mT/mG^-TRAMP transgenic mice (15w) (n=25, 5 separate mice for each genotype, 5 views per mouse). Scale bars, 50 μm. A Student’s t-test was performed for comparisons.

**Supplemental Figure 11. Prostate-specific Dach1 gene deletion in TRAMP mice induces mTOR signaling**. IPA pathway analysis of microarray data was conducted to compare Dach1 WT (*Dach1^wt/wt^; Pbsn-Cre; Rosa26^mT-mGfl/mTmGfl^; TRAMP*) versus Dach1 KO (*Dach1^fl/fl^; Pbsn-Cre; Rosa26^mT-mGfl/mTmGfl^; TRAMP*) PIN mice. P-values indicate significance of enrichment passing FDR<5% threshold. Z-scores calculated by IPA based on direction of change of pathway member genes where available indicate whether pathway is more active (positive Z-scores) or inhibited (negative Z-scores) in Dach1 WT vs Dach1 KO. mTOR was the number 4 most significantly affected pathway with 23 genes, p=1x10^-5^, and inhibited in Dach1 WT.

**Supplemental Figure 12. DACH1 facilitates recruitment of, and co-accumulates with, Ku70/Ku80 proteins at sites of DNA damage.** (A,C). Quantitation of co-accumulation of DACH1 and Ku-70/Ku-80 at laser micro irradiation-induced DSBs sites in *Dach1*^-/-^ 3T3 cells transfected with EGFP-tagged DACH1 and red fluorescent protein (RFP)-tagged Ku70 or RFP-tagged Ku80 expression vector or (B,D), control EGFP vector and RFP-tagged Ku70 or RFP-tagged Ku80 expression vector. Laser micro irradiation (403 nm) was used to induce DSBs 24 h after transfection. Accumulation of the transfected proteins was indicated by EGFP (green) or RFP (red) fluorescence at laser-irradiated sites in Fig. 6B-C. Time is shown in minutes and -fold increase in foci intensity is shown as mean ± SEM for N=5 separate cells.

**Supplemental Figure 13. DACH1 binds Ku70 and Ku80.** (A). Experimental approach used for identification of DACH1-binding proteins. HEK 293T cells transiently transfected with FLAG-DACH1-expressing vector. 50 mg of whole cell lysates were subjected to an immune-affinity column preloaded with a 1-ml slurry of M2 agarose beads (Sigma). The proteins associated with agarose beads were eluted with buffer containing 100 μm FLAG peptide [[3](#_ENREF_3)]. (B-C). Schematic representation of DACH1 expression vectors. 293T cells were transiently transfected with FLAG-DACH1 wild type, FLAG-DACH1 DS domain deletion (ΔDS), FLAG-DACH1 C term, or vector control. Two days later total cellular lysates were subjected to immunoprecipitation with DACH1 antibody and Western blot analysis. (D). immunoprecipitation-Western blot of DACH1-associated proteins. 293T cells were transiently transfected with FLAG-Ku70 + Myc-DACH1 or vector control. Two days later total cellular lysates were subjected to immunoprecipitation with FLAG antibody (for FLAG-Ku70) or DACH1 antibody and Western blot analysis. The antibodies used in (C) and/or (D): were rabbit polyclonal DACH1 antibody (Proteintech 10914-1-AP), rabbit polyclonal Ku80 antibody (Invitrogen PA517454), mouse monoclonal Ku70 antibody (Fisher MS329P), mouse monoclonal FLAG antibody (Sigma F3165), and rabbit polyclonal GAPDH antibody (sc-25778) as a loading control. (E). The potential kinases for human DACH1 Ser439 were analyzed using PhosphoNET Kinase Predictor, <http://www.phosphonet.ca/default.aspx?search=DACH1>. The potential kinases for Ser439 site of the motif IKERVPD(ph)SPSPAPSL (pSer439-DACH1) are shown, <http://www.phosphonet.ca/kinasepredictor.aspx?uni=Q9UI36&ps=S491>.

**Supplemental Figure 14. *Dach1^-/-^* cells are resistant to TGF-β receptor type I (TGF-βRI) kinase inhibitors in the presence of the DNA damaging agent Doxorubicin in cell growth assays.** (A). *Dach1*^-/-^ 3T3 transduced with a DACH1 expression vector were treated for 3 days with Doxorubicin (2 μM) and increasing doses of the TGF-β receptor type I (TGF-βRI) kinase inhibitors LY2157299, or (B). LY363947, or vehicle control. **Data are shown as mean** ± **SEM for N=3 separate experiments in triplicate.**

**Supplemental Figure 15.** ***Dach1^-/-^* cells are resistant to TGF-β receptor type I (TGF-βRI) kinase inhibitors in the presence of the DNA damaging agent Doxorubicin in comet assays.** (A). Schematic representation of the treatment protocol. *Dach1* WT and *Dach1* KO 3T3 cells were treated with TGF-β receptor type I (TGF-βRI) kinase inhibitor 20 μM LY2157299, or 1 μM LY363947, or vehicle control DMSO for 3 days. After treatment with 2 μM doxorubicin or control for 24 hours cells were harvested and processed for neutral pH Comet assay. (B). Representative examples of cellular comets for the cell types as shown. (C). Average tail moments were analyzed using OpenComet software. Data are the mean and standard error from 125-267 cells per treatment.

**Supplemental Figure 16.** **shDACH1 sensitizes prostate cancer cells to irradiation and DNA-PK inhibitor NU7026 in colony assays.** LNCaP cells transduced with *DACH1* shRNA or shRNA control vector were exposed to 4 Gy X-ray irradiation (RS 2000 Biological System irradiator, Rad Source, USA) followed by clonogenic assays to determine cell survival. Cells were pre-treated with 20 μM NU7026 for 24 hours before radiation. ImageJ software was used for quantification of colony number and size. The data are shown as mean and standard error from triplicates expressed as either (A) normalized survival fraction or (B) normalized colony size.

**REFERENCES**

1 Cancer Genome Atlas Research N. The Molecular Taxonomy of Primary Prostate Cancer. *Cell* 2015; 163: 1011-1025.

2 Liu J, Lichtenberg T, Hoadley KA, Poisson LM, Lazar AJ, Cherniack AD *et al*. An Integrated TCGA Pan-Cancer Clinical Data Resource to Drive High-Quality Survival Outcome Analytics. *Cell* 2018; 173: 400-416 e411.

3 Zhou J, Liu Y, Zhang W, Popov VM, Wang M, Pattabiraman N *et al*. Transcription elongation regulator 1 is a co-integrator of the cell fate determination factor Dachshund homolog 1. *J Biol Chem* 2010; 285: 40342-40350.
